# Sepsis 2016 Paris: Paris, France. 6-8 December 2016

**DOI:** 10.1186/s13054-016-1518-8

**Published:** 2016-12-06

**Authors:** Marcia Ingles, Gary Crowfoot, Tamara V. Smelaya, Artem N. Kuzovlev, Lubov E. Salnikova, Raisa Bhikoo, Bodin Khwannimit, Rungsun Bhurayanontachai, Veerapong Vattanavanit, Emilie Tourteau, Amel Filali, Nicolas van Grunderbeeck, Olivier Nigeon, Hélène Bazus, Juliette Masse, Jihad Mallat, Didier Thevenin, Isabella Prokhorenko, Dmitry Kabanov, Svetlana Zubova, Sergey Grachev, Margarita Salcedo, Stephan Witte, Valérie Cuvier, Marc Derive, Sébastien Gibot, Jean-Jacques Garaud, Vijay Kumar, Sanjay Chhibber, Jerico R. Santos, Jesus Emmanuel A. D. Sevillejal, Jose B. Nevado, Helena M. Linge, Kanta Ochani, Ke Lin, Ji Young Lee, Ping Wang, Manoj Tembhre, Shu Fang Liu, Pravin C. Singhal, Edmund J. Miller, Jeffery HO, Xiaodong Liu, Thomas Kwong, Lin Zhang, Hung Chan, Sunny H. Wong, Gordon Choi, Tony Gin, Matthew T. V. Chan, William K. K. Wu, Gwendolyn Vliegen, Kaat Kehoe, Robert Verkerk, Erik Fransen, Esther Peters, Anne-Marie Lambeir, Peter Pickkers, Philippe G. Jorens, Ingrid De Meester, Aline Barbosa Ribeiro, Ana Paula Trevelin Souza, Humberto Giusti, Celso Rodrigues Franci, Rafael Simone Saia, Renee R. Anderko, Vanessa M. Jackson, Octavia M. Peck Palmer, Derek C. Angus, John A. Kellum, Joseph A. Carcillo, D. M. Verboom, M. E. Koster-Brouwer, K. van de Groep, J. F. Frencken, B. Scicluna, S. S. Gisbertz, M. I. van Berge Henegouwen, J. P. Ruurda, R. van Hillegersberg, T. van der Poll, M. J. M. Bonten, O. L. Cremer, D. M. Verboom, J. F. Frencken, T. van der Poll, M. J. M. Bonten, O. L. Cremer, P. M. C. Klein Klouwenberg, Natalia Beloborodova, Artem Osipov, Alisa Pautova, Aleksandra Bedova, Jaime Mas-Oliva, Victor García-González, Marina Sukhina, Vladimir Zhukhovitskiy, Marina A. Sukhina, Igor Obraztsov, Vladimir G. Zhukhovitskiy, Cinzia Peronace, Giovanni Matera, Luisa Galati, Aida Giancotti, Giorgio Settimo Barreca, Angela Quirino, Maria Carla Liberto, Alfredo Focà, Yasmine Labiad, Geoffroy Venton, Céline Baier, Julien Colle, Laure Farnault, Corinne Brunet, Béatrice Loriod, Nicloas Fernandez-Nunez, Pierre Suchon, Jean-Camille Mattei, Pascal Rihet, Catherine Nguyen, Régis Costello, Abel Tesfai, Blerina Ahmetaj-Shala, Hime Gashaw, Gregory Quinlan, Niall MacCallum, Sharon Mumby, DNicola Gray, James Leiper, Nicholas Kirkby, Jane A. Mitchell, Luis Henrique A. Costa, Carlos Henrique R. Catalão, Nilton N. Santos-Júnior, Anderson O. Souza, Luciane C. Alberici, Maria José A. Rocha

**Affiliations:** 1 Belmont Hospital Emergency Department, Hunter New England Local Health District, New South Wales Health, New South Wales, Australia; 2 Main Military Clinical Hospital of Internal Troops of Russia, Balashikha, Moscow region Russia; 3 V. A. Negovsky Research Institute of General Reanimatology, Russian Academy of Sciences, Moscow, Russia; 4 N.I. Vavilov Institute of General Genetics, Russian Academy of Sciences, Moscow, Russia; 5 Department of Internal Medicine, Tygerberg Hospital, Cape Town, South Africa; 6 Division of Critical Care Medicine, Department of Internal Medicine, Faculty of Medicine, Prince Songkla University, Hat Yai, Songkhla 90110 Thailand; 7 Service de Réanimation Polyvalente & USC, Centre Hospitalier de Lens, Lens, France; 8 Service de Maladies Infectieuses, CHRU Lille, Lille, France; 9 Service d’Accueil des Urgences, Centre Hospitalier de Lens, Lens, France; 10 Service de Maladies Infectieuses, Centre Hospitalier de Lens, Lens, France; 11 FSBSI Institute of Basic Biological Problems RAS, Pushchino, Moscow region Russia; 12 SBEI HPE I.M. Sechenov’s First Moscow State Medical University of Russian’s Ministry of Healthcare, Moscow, Russia; 13 INOTREM SA, Nancy and Paris, France; 14 INSERM U1116, Faculté de Médecine de Nancy, Université de Lorraine, Nancy, France; 15 Medical Intensive Care Unit, Hôpital Central, CHU Nancy, Nancy, France; 16 Department of Microbiology, Panjab University, Chandigarh, India; 17 Department of Paediatrics and Child Health, Mater Research, School of Medicine, University of Queensland, Brisbane, Queensland Australia; 18 College of Medicine, University of the Philippines-Manila, Manila, Philippines; 19 Institute of Molecular Biology and Biotechnology, National Institutes of Health, University of the Philippines-Manila, Manila, Philippines; 20 Institute of Human Genetics, National Institutes of Health, University of the Philippines-Manila, Manila, Philippines; 21 Department of Biochemistry and Molecular Biology, College of Medicine, University of the Philippines-Manila, Manila, Philippines; 22 Heart and Lung Research Unit, The Feinstein Institute for Medical Research, Northwell Health, Manhasset, NY USA; 23 Department of Anaesthesia and Intensive Care, Prince of Wales Hospital, The Chinese University of Hong Kong, Shatin, Hong Kong; 24 Department of Medicine and Therapeutics, Prince of Wales Hospital, The Chinese University of Hong Kong, Shatin, Hong Kong; 25 Laboratory of Medical Biochemistry, University of Antwerp, Universiteitsplein 1, 2610 Wilrijk, Belgium; 26 StatUa Center for Statistics, University of Antwerp, Prins Boudewijnlaan 43, 2650 Edegem, Belgium; 27 Department of Intensive Care Medicine, Radboud university medical center, 6500HB Nijmegen, The Netherlands; 28 Department of Pharmacology and Toxicology, Radboud university medical center, 6500HB Nijmegen, The Netherlands; 29 Department of Critical Care Medicine, Antwerp University Hospital and Laboratory of Experimental Medicine and Pediatrics, University of Antwerp, Wilrijkstraat 10, 2650 Edegem, Belgium; 30 Department of Physiology, Ribeirão Preto Medical School, University of São Paulo, Ribeirão Preto, São Paulo Brazil; 31 Department of Critical Care Medicine, University of Pittsburgh School of Medicine, Pittsburgh, PA USA; 32 University of Pittsburgh Medical Center, Pittsburgh, PA USA; 33 Department of Pathology, University of Pittsburgh School of Medicine, Pittsburgh, PA USA; 34 Center for Critical Care Nephrology, University of Pittsburgh, Pittsburgh, PA USA; 35 Department of Pediatrics, University of Pittsburgh School of Medicine, Pittsburgh, PA USA; 36 Children’s Hospital of Pittsburgh of University of Pittsburgh Medical Center, Pittsburgh, PA USA; 37 Julius Center for health sciences and primary care, University Medical Center Utrecht, Utrecht, The Netherlands; 38 Department of Intensive Care, University Medical Center Utrecht, Utrecht, The Netherlands; 39 Center for Experimental and Molecular Medicine, Academic Medical Center, University of Amsterdam, Amsterdam, The Netherlands; 40 Department of Surgery, Academic Medical Center, Amsterdam, The Netherlands; 41 Department of Surgery, University Medical Center Utrecht, Utrecht, The Netherlands; 42 Center for Infection and Immunity, Academic Medical Center, Amsterdam, The Netherlands; 43 Division of Infectious Diseases, Academic Medical Center, Amsterdam, The Netherlands; 44 Department of Medical Microbiology, University Medical Center Utrecht, Utrecht, The Netherlands; 45 Julius Center for Health Sciences and Primary Care, University Medical Center Utrecht, Utrecht, The Netherlands; 46 Department of Intensive Care, University Medical Center Utrecht, Utrecht, The Netherlands; 47 Center for Infection and Immunity, Academic Medical Center, Amsterdam, The Netherlands; 48 Division of Infectious Diseases, Academic Medical Center, Amsterdam, The Netherlands; 49 Department of Medical Microbiology, University Medical Center Utrecht, Utrecht, The Netherlands; 50 Negovsky Research Institute of General Reanimatology, Moscow, Russia; 51 Instituto de Fisiología Celular, Universidad Nacional Autónoma de México, Cd. de México, México; 52 Facultad de Medicina, Universidad Autónoma de Baja California, Mexicali, México; 53 Department for Microbiology and Immunology, A.N. Ryzhikh State Scientific Center for Coloproctology, Moscow, Russia; 54 Laboratory for Identification and Analysis of Microorganisms, N.F. Gamalei State Research Centre for Epidemiology and Microbiology, Moscow, Russia; 55 Department for Microbiology and Immunology, A.N. Ryzhikh State Scientific Center for Coloproctology, Moscow, Russia; 56 Department for Clinical Immunology, D. Rogachev State Scientific and Clinical Center for Paediatric Haematology, Oncology and Immunology, Moscow, Russia; 57 Laboratory for Identification and Analysis of Microorganisms, N.F. Gamalei State Research Centre for Epidemiology and Microbiology, Moscow, Russia; 58 Institute of Microbiology, Department of Health Sciences, University “Magna Graecia” of Catanzaro, Catanzaro, Italy; 59 Aix-Marseille Université ; INSERM, UMR1090, TAGC Campus de Luminy, Marseille, F_13288 France; 60 Service d’Hématologie et de Thérapie Cellulaire Assistance Publique des Hôpitaux de Marseille, Centre Hospitalier Universitaire La Conception, Marseille, France; 61 Laboratoire d’Hématologie, Assistance Publique des Hôpitaux de Marseille, Centre Hospitalier Universitaire La Timone, Marseille, France; 62 UMR 1062 NORT, INSERM, Marseille, France; 63 UMR-911 INSERM Center for research in onco-biology and onco-pharmacology, faculté de médecine, Marseille, France; 64 National Heart & Lung Institute, Royal Brompton Campus, Faculty of Medicine, Imperial College London, SW3 6LY London, England; 65 Department of Biomolecular Medicine, Sir Alexander Fleming Building, South Kensington Campus, Faculty of Medicine, Imperial College London, SW7 2AZ London, England; 66 MRC Clinical Sciences Centre, Hammersmith Hospital, Du Cane Road, London, W12 0NN UK; 67 Department of Neurosciences and Behavioral Sciences, Ribeirão Preto Medical School, University of São Paulo, Ribeirão Preto, Brazil; 68 Department of Physics and Chemistry, School of Pharmaceutical Sciences of Ribeirão Preto, University of São Paulo, Ribeirão Preto, SP Brazil; 69 Department of Morphology, Physiology and Basic Pathology, School of Dentistry of Ribeirão Preto, University of São Paulo, Ribeirão Preto, Brazil

## P1 Reduction in time to first dose antibiotics in one Australian Emergency Department

### Marcia Ingles, Gary Crowfoot

#### Belmont Hospital Emergency Department, Hunter New England Local Health District, New South Wales Health, New South Wales, Australia

##### **Correspondence:** Marcia Ingles (marcia.ingles@hnehealth.nsw.gov.au)

**Background**

**Fig. 1 (abstract P1).**
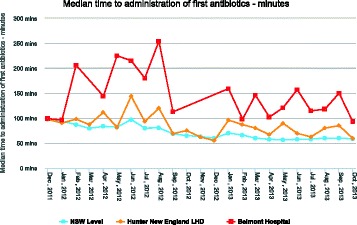
See text for description

Sepsis affects over 26 million people worldwide each year resulting in a death every 3 to 4 seconds [1]. For every hour that antibiotics are delayed after the first episode of hypotension, there is a 7.6 % increase in the risk of mortality [2]. Thus, international sepsis guidelines recommend the administration of broad spectrum antimicrobial therapy within 1 hour of recognition [3]. In 2011, the New South Wales Clinical Excellence Commission (CEC) developed the Sepsis Kills program including the *Adult Emergency Sepsis Pathway* [4]. This pathway was introduced to Emergency Departments (ED) and auditing of time to first antibiotics commenced. Belmont Hospital Emergency Department has approximately 25000 presentations per year. In 2012, 163 patients were diagnosed with sepsis. Time to first antibiotics for sepsis patients peaked at 254 minutes (Fig. 1). Discussion of these results highlighted the need to develop education strategies in order to reduce time to first administration of antibiotics.

**Materials and methods**

Audits of the CEC Sepsis database [5] over 4 years included a total of 769 patients. Collected data entered included: age, triage time and category, clinical observations, time and amount of intravenous fluid, time to first antibiotics, and diagnosis. The tables provided reflect median time to antibiotics. In 2012 a Clinical Nurse Specialist from the ED was designated Sepsis Lead. Over 4 years, the Sepsis Lead worked collaboratively with clinical staff to develop and implement several strategies to decrease time to first antibiotics

**Results**

**Fig. 2 (abstract P1).**
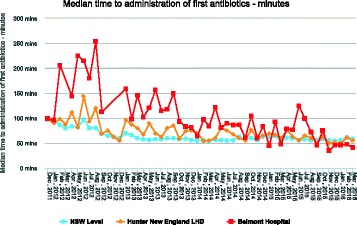
See text for description

A multimodal approach strategy was adopted, which included: regular audits; targeted education programs for triage nurses and nursing team leaders; the introduction of Sepsis September - a month dedicated to sepsis awareness and education; and the Sepsis Road show. These interventions were well received by Emergency staff. As a direct result time to first antibiotics was reduced to a median of 41 minutes (Fig. 2).

**Conclusions**

The collaborative effort between the Sepsis Lead and clinical staff has produced a significant reduction in time to first antibiotics from 254 minutes to 41 minutes at last audit. The success achieved at Belmont Hospital due to this multimodal approach strategy has the potential to be translated globally. It also serves to highlight the importance of the Emergency Nurse in early recognition and initiation of treatment for sepsis.

**References**

1. Gigliotti E, Steele J, Cassidy D, Bell-Gordon CR: The development and implementation of a nurse practitioner sepsis screening team: Impact on transfer mortality. Journal of Nursing Education and Practice 2014, 4(6):77–83

2. Clinical Excellence Commission: Sepsis Kills Program [http://www.cec.health.nsw.gov.au/programs/sepsis]

3. Dellinger RP, Levy MM, Rhodes A, Annane D, Gerlach H, Opal SM, Sevransky J, Sprung CL, Douglas IS, Jaeschke J, et al.: Surviving Sepsis Campaign: International Guidelines for Management of Severe Sepsis and Septic Shock, 2012. Intensive Care Med 2013, 39:165–228

4. Clinical Excellence Commission: Sepsis Tools [http://www.cec.health.nsw.gov.au/programs/sepsis/sepsis-tools#Pathways]

5. Clinical Excellence Commission: Sepsis Database [http://www.cec.health.nsw.gov.au/]

## P2 The search for genetic markers in the development of acute respiratory failure

### Tamara V Smelaya^1,2^, Artem N Kuzovlev^2^, Lubov E Salnikova^2,3^

#### ^1^Main Military Clinical Hospital of Internal Troops of Russia, Balashikha, Moscow region, Russia; ^2^V. A. Negovsky Research Institute of General Reanimatology, Russian Academy of Sciences, Moscow, Russia; ^3^N.I. Vavilov Institute of General Genetics, Russian Academy of Sciences, Moscow, Russia

##### **Correspondence:** Artem N Kuzovlev (artem_kuzovlev@mail.ru)

**Background**

Despite advances in diagnostic methods and antibiotic therapy, community-acquired pneumonia (CAP) is still a major cause of morbidity and mortality worldwide. Risk of CAP has been attributed to pathogen virulence, host susceptibility and epidemiologic factors. A significant number of patients with CAP develop severe complications, such as sepsis, acute respiratory distress syndrome (ARDS), multiple organ dysfunction syndrome (MODS) and less fatal conditions (pleuritis, empyema) and syndromes (acute respiratory failure (ARF)). The variable clinical presentation of CAP suggests a genetic predisposition.

**Materials and methods**

This study was conducted to establish the possible contribution of functional gene polymorphisms in the oxidative stress related genes to the development of community-acquired pneumonia (CAP) complications. CAP subjects (n = 350) were genotyped for 16 polymorphic variants in the genes of xenobiotics detoxification CYP1A1, AhR, GSTM1, GSTT1, ABCB1, redox-status SOD2, CAT, GCLC, and vascular homeostasis ACE, AGT, AGTR1, NOS3, MTHFR, VEGFa.

**Results**

The multilocus model which included six or more risk alleles in the CYP1A1, GCLC, AGT and AGTR1 genes was associated with pleuritis, empyema, acute respiratory distress syndrome (ARDS), all pulmonary complications together and acute respiratory failure. Genetically mediated correlation between clinical conditions in CAP patients is shown in Table 1. In silico analysis with Set Distiller mode identified N-acetylcysteine (P = 1.08E-08) and oxygen (P = 1.92E-06) as the best descriptors for the considered gene set. Acute infections of the airways are associated with oxidative stress, which enhances viscosity of bronchial mucus, reduces the mucociliary clearance rate and expedites lung disease aggravation and progression. N-acetylcysteine is a well-known mucolytic and antioxidant drug, an indirect precursor of glutathione.

**Conclusions**

The results of the study indicate that pneumonia aggravation up to destructive intrapulmonary complications and ARDS is mediated by polymorphisms in oxidative stress-related genes.

**Table 1 (abstract P2).** Correlation between clinical conditions in stratified analysis based on the cumulative gene risk score

Number of the risk alleles in the genes,	0-5 risk alleles	6-9 risk alleles
*CYP1A1* (rs2606345 - T, rs4646903 - T,		
rs1048943 - A),		
*GCLC* (rs17883901 - T)*,*		
*AGT* (rs699 - C)*,*		
*AGTR1* (rs5186 - C)		
PC - ARF	r = 0.43 (*P* = 2.4E-07)	r = 0.57 (*P* = 2.1E-18)
PSI - ARF	r = 0.60 (P = 1.6E-06)	r = 0.67 (P = 3.0E-16)

*PC* pulmonary complications, *ARF* acute respiratory failure, *PSI* pneumonia severity index, *r* correlation coefficient, *P P*- value at two sided significance level

## P3 A retrospective study evaluating the efficacy of identification and management of sepsis at a Western Cape Province district level hospital internal medicine department, in comparison to the guidelines stipulated in the Surviving Sepsis Campaign 2012

### Raisa Bhikoo (raisa3121@gmail.com)

#### Department of Internal Medicine, Tygerberg Hospital, Cape Town, South Africa

**Background**

Currently there is little data on identification, management and outcomes of patients with sepsis in developing countries. In low income countries major concerns regarding accessibility to healthcare, limitations due to costs, lack of resources and delayed presentations of patients with sepsis make implementing protocols, based on developed countries patients profile difficult. Thus in Sub-Saharan Africa there has been a widespread shift towards protocol development that is cost effective and specific for the epidemiologic and ecologic data.

**Materials and methods**

The aim of our study is to assess the efficacy of clinicians at a district level hospital in the Western Cape at identifying and managing sepsis. Furthermore, we will assess the outcome of patients in terms of in-hospital mortality and length of hospital stay given the above management. A retrospective study design was applied when analyzing data from the routine burden of disease audit done on a 3 monthly basis at Karl Bremer Hospital.

**Results**

The total sample size obtained was 70 patients. A total of 18/70 (26 %) patients had an initial triage blood pressure indicative of sepsis induced hypotension however only 1/18 (5.5 %) of these patients received an initial crystalloid fluid bolus of 30 ml/kg. The median time for antibiotic administration in septic shock was 4.65 hours. Further a positive delay in antibiotic administration (p value = 0.0039) was demonstrated. A total of 7/70 patients received no antibiotics in the first 24 hours of hospital admission. Of these 7, 3 of these patients could be classified as septic shock at presentation. All 3 patients died within 36 hours of hospital admission. The data showed 8/12 (66 %) of patients with septic shock received inappropriate amounts of fluids. The in-hospital mortality for sepsis was found to be 4/24 (17 %), for severe sepsis 11/34 (32 %) and a staggering 9/12(75 %) for septic shock. A positive association between in-hospital mortality and the following was found:

Time to first dose antibiotic administration (OR = 1.07, P value = 0.027, 95%CI = 1.008-1.14). For every 1-hour delay in antibiotic administration the chance of death increased by 7 %.

Source appropriate antibiotics (OR = 0.17, P value = 0.005, 95 % CI = 0.048-0.59). The chance of death amongst patients that received source appropriate antibiotics is 83 % less than those who did not.

Early appropriate intravenous fluid administration (OR = 0.33, P value = 0.040,95 % CI = 0.11-0.95). Appropriate intravenous fluids was associated with a 67 % reduction in in-hospital mortality.

**Conclusion**

The outcomes of the study concluded that there is room for improvement by our clinicians with regards to appropriate identification and management of patients with sepsis. Flawed management has inevitably shown to have an impact on in-hospital mortality. Simple, cost effective measures that can implemented regardless of resources include early appropriate antibiotics and early aggressive fluid therapy; with the potential for an inordinate impact on mortality.

**Acknowledgements**

Dr Sarah Versfeld: MBChb (Stell). Medical officer in the Department of Internal Medicine, Karl Bremer Hospital.

versfeld.sarah@gmail.com

Dr MMDe V Basson: MBChb (Stell), Hons BSc in Epidemiology (Stell), MMed in Internal Medicine (Stell).

Head of Internal Medicine and senior consultant at Karl Bremer Hospital. DeVries.Basson@westerncape.gov.za

Dr Almero H Oosthuizen: MBChb (Stell) (cum laude), Dip.PEC (SA), MMed EM (UCT) FCEM (SA). Senior consultant in the Department of Emergency Medicine, Karl Bremer Hospital.

almero.oosthuizen@westerncape.gov.za

## P4 Validation of the Sepsis Severity Score compared with standard severity scores in predicting hospital mortality in severe sepsis and septic shock patients

### Bodin Khwannimit, Rungsun Bhurayanontachai, Veerapong Vattanavanit

#### Division of Critical Care Medicine, Department of Internal Medicine, Faculty of Medicine, Prince of Songkla University, Hat Yai, Songkhla, 90110 Thailand

##### **Correspondence:** Bodin Khwannimit (kbordinbodin@gmail.com)

**Background**

Recently, the Sepsis Severity Score (SSS) [1] was constructed to estimate the probability of hospital mortality in severe sepsis and septic shock patients. The aim of this study was to validate and compare the performance of the SSS with the Acute Physiology and Chronic Health Evaluation (APACHE) II-IV [2], Simplified Acute Physiology Score (SAPS) II and SAPS 3 scores in predicting hospital outcome in sepsis patients.

**Materials and methods**

A retroprospective analysis was conducted of prospectively collected data from all consecutive sepsis patients admitted to the medical intensive care unit of a tertiary university teaching hospital. The performance of the severity scores was evaluated by discrimination, calibration and overall performance.

**Results**

**Fig. 3 (abstract P4).**
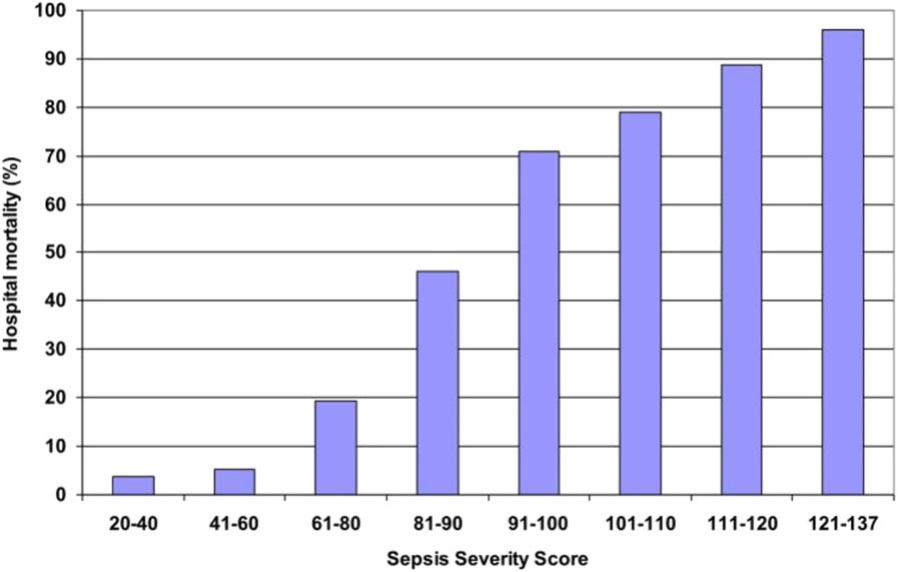
See text for description

From a total of 931 patients, 802 patients (87.8 %) who had septic shock were enrolled. The hospital mortality rate was 43.9 % and the mean age was 59.3 ± 21.5 years. The most common sources of ICU admission were from the emergency room (47.5 %), general wards (43.9 %), and transfers from other hospitals (8.6 %). Mechanical ventilation was used in 808 patients (88.5 %). Community-acquired infections accounted for 70.2 %. The most common sources of infection were respiratory (49.1 %), gastrointestinal (14.8 %) and primary bloodstream infections (9.9 %). The median SSS was 80 (range 20–137). Hospital mortality by the SSS is demonstrated in Fig. 3.

The standard mortality ratios of observed mortality to predicted mortality were between 0.81 and 1.1. The SSS presented good discrimination with an area under the receiver operating characteristic curve (AUC) of 0.892. The performances of each severity scoring system are shown in Table 2.

**Fig. 4 (abstract P4).**
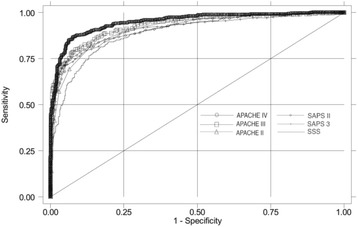
See text for description

**Fig. 5 (abstract P4).**
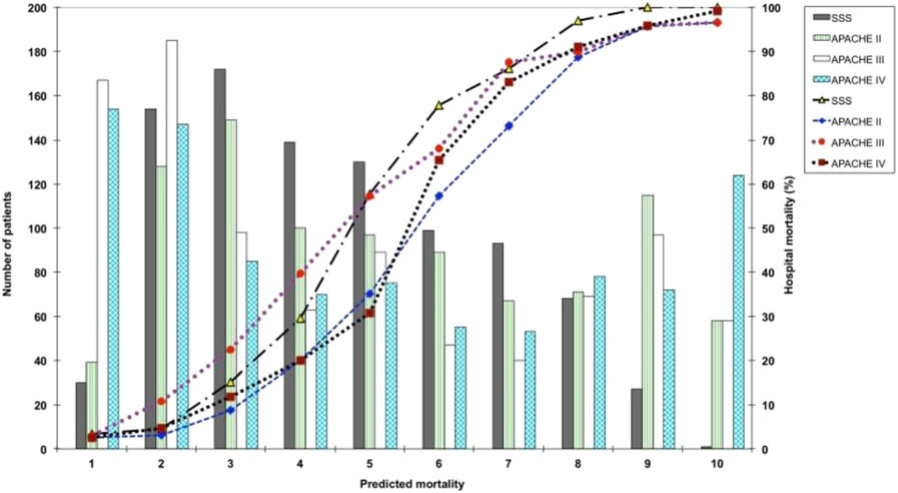
See text for description

The discriminative performance of the SSS was not statistically significant from the APACHE II (P = 0.07), SAPS II (P = 0.06) or SAPS 3 (P = 0.11). However, the APACHE IV score showed the best discrimination with an AUC of 0.948 and the overall performance by a Brier score of 0.096. The AUC of the APACHE IV score was statistically greater than the SSS (P < 0.0001), APACHE II (P < 0.0001), APACHE III (P = 0.0002), SAPS II (P < 0.0001) and SAPS 3 (P < 0.0001) (Fig. 4). The calibration of all scores was poor (Hosmer-Lemeshow goodness-of-fit H test <0.05). The calibration curves for the SSS and APACHE scores are presented in Fig. 5.

**Conclusions**

The SSS provided good discrimination as well as the APACHE II, SAPS II and SAPS 3 scores. However, the APACHE IV score had the best discrimination and overall performance in our severe sepsis and septic shock patients. The SSS needs to be adapted and modified with new parameters to improve the performance.

**References**

1. Osborn TM, Phillips G, Lemeshow S, Townsend S, Schorr CA, Levy MM, et al. Sepsis severity score: an internationally derived scoring system from the surviving sepsis campaign database. Crit Care Med. 2014:1969–1976.

2. Zimmerman JE, Kramer AA, McNair DS, Malila FM. Acute Physiology and Chronic Health Evaluation (APACHE) IV: hospital mortality assessment for today's critically ill patients. Crit Care Med. 2006:1297–1310.

**Table 2 (abstract P4).** Performance of the SSS, APACHE II, III, IV, SAPS II and SAPS 3 scores

	Score	Predicted	AUC	SMR	H	C	Brier score
	(mean ± SD)	mortality	(95 % CI)	(95 % CI)			
SSS	82.7 ± 22.9	39.9 ± 20.8	0.892	1.09	95.3*	36.3*	0.183
			(0.871-0.913)	(0.99-1.21)			
APACHE II	22.8 ± 9.7	47.8 ± 27.1	0.913	0.92	67.9*	10.8***	0.125
			(0.895-0.931)	(0.83-1.01)			
APACHE III	88.2 ± 40.1	39.7 ± 30	0.931	1.1	43.6*	11.5***	0.120
			(0.914-0.948)	(1.00-1.22)			
APACHE IV	-	45 ± 32	0.948	0.97	68.1*	6.2***	0.096
			(0.934-0.962)	(0.88-1.07)			
SAPS II	54.2 ± 22.8	51 ± 32.8	0.913	0.86	57.8*	22.3**	0.124
			(0.894-0.932)	(0.78-0.95)			
SAPS 3	71.6 ± 19.3	54 ± 28.1	0.909	0.81	114.2*	49.2*	0.139
			(0.889-0.929)	(0.73-0.9)			

*P < 0.0001, **P < 0.05, ***P > 0.05

*APACHE* Acute Physiology and Chronic Health Evaluation, *AUC* Area under the receiver operating characteristic, *C* Hosmer-Lemeshow goodness-of-fit C test, *CI* confidence interval, *H* Hosmer-Lemeshow goodness- of-fit H test, *SAPS* Simplified Acute Physiology Score, *SD* standard deviation, *SMR* standardized mortality ratio, *SSS* Sepsis Severity Score

## P5 What did hospital physicians know about Sepsis before sepsis 3.0? A monocentric survey amongst 132 physicians

### Emilie Tourteau^1^, Amel Filali^2^, Nicolas van Grunderbeeck^1,4^, Olivier Nigeon^1,3^, Hélène Bazus^4^, Juliette Masse^3^, Jihad Mallat^1^, Didier Thevenin^1^

#### ^1^Service de Réanimation Polyvalente & USC, Centre Hospitalier de Lens, Lens, France;^2^Service de Maladies Infectieuses, CHRU Lille, Lille, France; ^3^Service d'Accueil des Urgences, Centre Hospitalier de Lens, Lens, France; ^4^Service de Maladies Infectieuses, Centre Hospitalier de Lens, Lens, France

##### **Correspondence:** Nicolas van Grunderbeeck (nicovgdb9@orange.fr)

**Background**

Sepsis in a public health problem and septic patients suffer high mortality[1,2]. Few data are available about physicians' knowledge and capacity to manage sepsis outside the Intensive Care Unit (ICU) [3]. The physicians' knowledge about sepsis could explain discrepancies in management between Emergency Department (ED) and ward septic patients, who suffer a poorer quality of care and a poorer outcome[4]. We aimed to assess knowledge and ability to handle sepsis management through a questionnaire.

**Materials and methods**

Observational, prospective, monocentric study during 6 months (May to December 2015). A 26 questions sheet was distributed to all physicians. Questionnaires were anonymous, included age, experience and specialty. Questions involved definitions, epidemiology, outcome, diagnosis and management of severe sepsis/septic shock. Eight questions out of 25 were core questions, and a 26th evaluated physicians' self-appreciation of ability. Evaluation included levels of good, wrong answers (WA) or "no knowledge" (NK) with specialties and self-estimated ability. Physicians were compared as fellows or senior, and specialties between "referent" (ED or Anesthesiologists/Intensivists vs others). Statistical analysis was performed though Chi2, Fisher exact, or Mann–Whitney tests.

**Results**

A hundred and thirty-two physicians (48.9 %), answered to the questionnaire: 34 ED or ICU/anesthesia physicians (26 %), 82 medical specialists (62 %), 16 surgeons (12 %). Forty-five percent were fellows. Median age was 29 years, median experience was 1 year after diploma. Referent physicians expressed higher self-confidence (97 vs 52 %, p < 0.001). Nearly all of them knew about existence of international guidelines (97 %), whereas a third of others did not (68 %, p = 0.001). Surgeons had poorer results and regarded themselves as unable to handle sepsis. Fellows and senior physicians' knowledge were equal (11 vs 10,8 % of wrong answers). Core questions revealed flaws about definitions (22 % WA), sepsis outside the ICU (29 % WA/NK), possible severity without hypotension (40.5 % WA/NK), importance of lactatemia (24 % WA/NK), existence of guidelines (24 % WA/NK), delay of antibiotic treatment (78 % agreed for up to a 6 h delay). Knowledge about fluid expansion without hypotension was 88,5 %, but 32 % failed about fluid choice.

**Conclusions**

Our study presents limits: it's monocentric with a small sample, with no differences between intensivists or anesthesiologists, and some questions may have been difficult to interpret. Moreover, it was "medical-limited" and did not evaluate the nurses’ teams, a key factor. Nevertheless, it provides arguments to think that management of sepsis could be improved through better knowledge outside the ICU, that could trigger prompt recognition and management, or call to specialized teams.

**References**

1. Angus DC, van der Poll T. Severe sepsis and septic shock. N Engl J Med. 2013 Nov 21;369(21):2063.

2. Gaieski DF, Edwards JM, Kallan MJ, Carr BG. Benchmarking the incidence and mortality of severe sepsis in the United States. Crit Care Med 2013;41 (5):1167–74.

3. Djurkovic S, Baracaldo JC, Guerra JA, Sartorius J, Haaupt MT. A survey of clinicians addressing the approach to the management of severe sepsis and septic shock in the United States. J Crit Care 2010 Dec; 25(4):658.e1-6.

4. Esteban A, Frutos-Vivar F, Ferguson ND, Peñuelas O, Lorente JA, Gordo F, Honrubia T, Algora A, Bustos A, García G. Sepsis incidence and outcome: contrasting the intensive care unit with the hospital ward. Crit Care Med. 2007 May;35(5):1284–9.

## P6 CD14 of human polymorphonuclear leukocytes in endotoxin priming for respiratory burst

### Isabella Prokhorenko^1^, Dmitry Kabanov^1^, Svetlana Zubova^1^, Sergey Grachev^1,2^

#### ^1^FSBSI Institute of Basic Biological Problems RAS, Pushchino, Moscow region, Russia; ^2^SBEI HPE I.M. Sechenov’s First Moscow State Medical University of Russian’s Ministry of Healthcare, Moscow, Russia

##### **Correspondence:** Isabella Prokhorenko (kabanovd1@rambler.ru)

**Background**

The induction of inflammatory responses by lipopolysaccharides (LPSs, endotoxins) is achieved by the coordinate and sequential action of four principal LPS-binding proteins: LPS-binding protein (LBP), CD14, Toll-like receptor 4 (TLR4) and Myeloid differentiation protein – MD-2 [1]. CD14, the cell surface receptor of monocytes and neutrophils, is required for LPS activation of phospholipases, protein tyrosine kinases, protein kinases A and C as well as MAP kinases [2]. TLR4 independently of CD14 cannot mobilize all of the adapter proteins that it requires for full signaling activity [3]. CD14 functional multiplicity provides the reason for their implication in target therapy of inflammatory response to LPS by development of blocking monoclonal antibodies (mAbs) specific to human CD14 [4] or TLR4 [5]. In the present study we have examined CD14 involvement in endotoxin priming of human polymorphonuclear leukocytes (PML) for respiratory burst triggered by N-formyl-methionyl-leucyl-phenylalanine (fMLP).

**Materials and methods**

Human PML were isolated from heparinized blood of healthy volunteers by standard procedure and incubated with or without anti-CD14 mAbs UCHM-1 or isotype-matched IgG2a for 30 min before priming by LPS. Control cells and cells that had been preexposed to mAbs or IgG2a were placed into chemiluminometer chambers containing solution for luminol-enhanced chemiluminescence (CL) measurement supplemented with autologous serum (2 %) and 0.01 mM CaCl2. PML priming was achieved by addition of 100 ng/ml S- or Re-LPS from Escherichia coli O55:B5 or JM103 followed by continuous gentle shaking for 30 min at 37 °C. CL had been triggered by adding of 1 μM fMLP and the light emission was recorded continuously for 20 min. Statistical significance was determined by applying the Wilcoxon’s signed-rank date analysis. Differences were considered to be significant when p < 0.05.

**Results**

**Fig. 6 (abstract P6).**
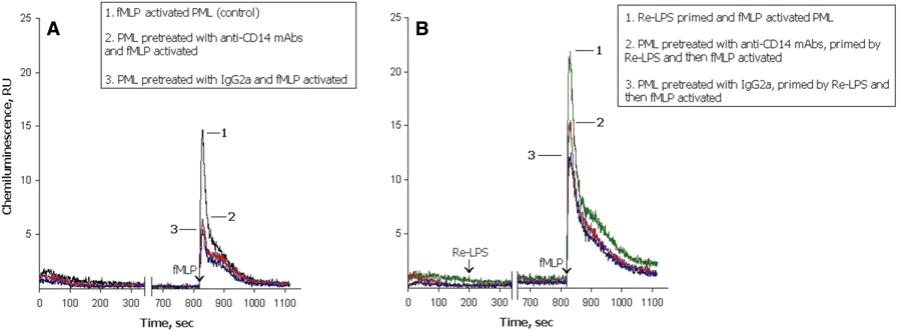
Effect of anti-CD14 mAbs or IgG2a on fMLP-triggered respiratory burst of control unprimed (**a**) and Re-LPS primed human PML (**b**)

**Fig. 7 (abstract P6).**
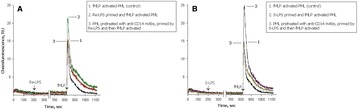
Comparison of the anti-CD14 mAbs effects on fMLP-triggered respiratory burst of human PML primed by Re-LPS (**a**) or S-LPS (**b**)

LPS is known to be engaged by CD14 and then transferred to TLR4 [2, 6, 7]. To assess CD14 involvement in endotoxin priming of human PML for fMLP-triggered respiratory burst we used the full anti-CD14 mAbs containing Fc-regions. We have therefore examined the effect of anti-CD14 mAbs as well as IgG2a on fMLP-triggered respiratory burst of control LPS-unprimed PML. As described in Fig. 6a incubation of PML with anti-CD14 mAbs decreased the level of fMLP-triggered CL caused by reactive oxygen species (ROS) production (p < 0.05). The same pattern was recorded when isotype-matched IgG2a had been employed (p < 0.05) (Fig. 6a). It is not yet known whether this inhibitory effect of the IgG2a’s Fc-regions on fMLP-triggered ROS generation will be still maintained during PML priming by endotoxins. When PML had been exposed to anti-CD14 mAbs as well as to IgG2a and then primed by Re-LPS near the same suppression of fMLP-triggered CL was detected (p < 0.05) (Fig. 6b). Compared to the control cells (75.29 ± 23.4 RU) the average value of fMLP-triggered CL in Re-LPS primed PML was increased up to 161.77 ± 49.4 RU (p < 0.05). However, after exposition of PML to anti-CD14 mAbs or IgG2a followed by Re-LPS priming the fMLP-triggered CL was reduced up to 112.55 ± 34.3 RU (by 30 %) or up to 126.90 ± 17.6 RU (by 22 %), respectively (Fig. 6b). Since “quenching” effects of Fc-regions of full anti-CD14 mAbs as well as isotype-matched IgG2a on fMLP-triggered respiratory burst have been almost identical it is not possible at the moment to make decision what is the real impact of F(ab')2-regions of used anti-CD14 mAbs in PML priming by LPS. Some indication on the impact of F(ab')2-regions in this process may came from the comparison of anti-CD14 mAbs effect on PML priming by different LPS-glycoforms, namely S- or Re-LPS. As follows from Fig. 7a and b the most pronounced inhibitory effect of anti-CD14 mAbs on PML priming by S-LPS from E. coli has been achieved (by 40 %). Especially role of CD14 in delivery of S-LPS to TLR4 is well described [8].

**Conclusions**

LPS is known to be engaged by CD14 and then transferred to TLR4 [2, 6, 7]. To assess CD14 involvement in endotoxin priming of human PML for fMLP-triggered respiratory burst we used the full anti-CD14 mAbs containing Fc-regions. We have therefore examined the effect of anti-CD14 mAbs as well as IgG2a on fMLP-triggered respiratory burst of control LPS-unprimed PML. As described in Fig. 6a incubation of PML with anti-CD14 mAbs decreased the level of fMLP-triggered CL caused by reactive oxygen species (ROS) production (p < 0.05). The same pattern was recorded when isotype-matched IgG2a had been employed (p < 0.05) (Fig. 6a). It is not yet known whether this inhibitory effect of the IgG2a’s Fc-regions on fMLP-triggered ROS generation will be still maintained during PML priming by endotoxins. When PML had been exposed to anti-CD14 mAbs as well as to IgG2a and then primed by Re-LPS near the same suppression of fMLP-triggered CL was detected (p < 0.05) (Fig. 6b). Compared to the control cells (75.29 ± 23.4 RU) the average value of fMLP-triggered CL in Re-LPS primed PML was increased up to 161.77 ± 49.4 RU (p < 0.05). However, after exposition of PML to anti-CD14 mAbs or IgG2a followed by Re-LPS priming the fMLP-triggered CL was reduced up to 112.55 ± 34.3 RU (by 30 %) or up to 126.90 ± 17.6 RU (by 22 %), respectively (Fig. 6b). Since “quenching” effects of Fc-regions of full anti-CD14 mAbs as well as isotype-matched IgG2a on fMLP-triggered respiratory burst have been almost identical it is not possible at the moment to make decision what is the real impact of F(ab')2-regions of used anti-CD14 mAbs in PML priming by LPS. Some indication on the impact of F(ab')2-regions in this process may came from the comparison of anti-CD14 mAbs effect on PML priming by different LPS-glycoforms, namely S- or Re-LPS. As follows from Fig. 7a and b the most pronounced inhibitory effect of anti-CD14 mAbs on PML priming by S-LPS from E. coli has been achieved (by 40 %). Especially role of CD14 in delivery of S-LPS to TLR4 is well described [8].

**References**

1. Peri F, Piazza M: Therapeutic targeting of innate immunity with Toll-like receptor 4 (TLR4) antagonist. Biotechnology Advances 2012, 30: 251–260.

2. Yan S, Al-Hertani W, Byers D, Bortolussi R: Lipopolysaccharide-binding protein- and CD14-dependent activation of mitogen-activated protein kinase p38 by lipopolysaccharide in human neutrophils is associated with priming of respiratory burst. Infect Immun 2002, 70: 4068–4074.

3. Beutler B, Jiang Z, Georgel P, Crozat K, Croker B, et al.: Genetic analysis of host resistance: Toll-like receptor signaling and immunity at Large. Annu Rev Immunol 2006, 24: 353–389.

4. Verbon A, Dekkers P, Tessa ten Hove, Hack C, Pribble J, et al.: IC14, an anti-CD14 antibody, inhibits endotoxin-mediated symptoms and inflammatory responses in humans. J Immunol 2001, 166: 3599–3605.

5. Dunn-Siegrist I, Leger O, Daubeuf B, Poitevin Y, Depis F, et al.: Pivotal involvement of Fcγ receptor IIA in the neutralization of lipopolysaccharide signaling via a potent novel anti-TLR4 monoclonal antibody 15C1. J Biol Chem 2007, 282: 34817–34827.

6. Golenbock D, Liu Y, Millham F, Freeman M, Zoeller R: Surface expression of human CD14 in Chinese hamster ovary fibroblasts imparts macrophage-like responsiveness to bacterial endotoxin. J Biol Chem 1993, 268: 22055–22059.

7. Plociennikowska A, Hromada-Judycka A, Borzecka K, Kwiatkowska K: Co-operation of TLR4 and raft proteins in LPS-induced pro-inflammatory signaling. Cell Mol Life Sci 2015, 72: 557–581.

8. Triantafilou M, Triantafilou K, Fernandez N: Rough and smooth forms of fluorescein-labelled bacterial endotoxin exhibit CD14/LBP dependent and independent binding that is influenced by endotoxin concentration. Eur J Biochem 2000, 267: 2218–2226.

## P7

**Withdrawn**

## P8 Clarithromycin exerts protective immunomodulatory action during *Klebsiella pneumoniae* B5055 induced pneumonia in BALB/c mice

### Vijay Kumar^1,2^, Sanjay Chhibber^1^

#### ^1^Department of Microbiology, Panjab University, Chandigarh, India; ^2^ Department of Paediatrics and Child Health, Mater Research, School of Medicine, University of Queensland, Brisbane, Queensland, Australia

##### **Correspondence:** Vijay Kumar (vij_tox@yahoo.com)

**Background**

Bacterial pneumonia is a life threatening condition causing acute lung injury (ALI) leading to development of sepsis depending on the invasiveness of the bacteria and host immune status. This present study was designed to find out the protective immunomodulatory action of macrolide antibiotic (i.e. clarithromycin) in mouse model of pneumonia induced by *K. pneumoniae* B5055.

**Materials and Methods**

Pneumonia was induced by intranasal instillation of *Klebsiella pneumoniae* B5055 (10^4^ cfu/ml) by holding the mice in upright position without any anaesthesia. Experimental animals were divided in two groups (i.e. Group A; control (oral saline), Group B; clarithromycin (30 mg/kg/day/os). At designated period of time (1, 2, 3, 5 and 7 days) mice were euthanized by cervical dislocation and lungs were harvested and homogenized in 1 ml of sterile normal saline under sterile conditions. The lung homogenate was used for measuring bacterial load, malondialdehyde (MDA), nitric oxide (NO) and myeloperoxidase (MPO) and pro-inflammatory (i.e. IL-1α, IL-6, TNF-α) anti-inflammatory (i.e. IL-10) cytokine levels in lungs. Histopathological examination of the lungs was also carried out along with lung alveolar macrophage function (i.e. macrophage spreading and phagocytosis) assays.

**Results**

Clarithromycin treatment led to the significant (p < 0.05) reduction in pulmonary neutrophil infiltration along with significant (p < 0.05) decrease in other markers of inflammation (TNF-α, IL-α, IL-6, MDA, NO and MPO) associated with acute tissue injury or inflammation. The increase in bacterial clearance in lungs of mice treated with clarithromycin was associated with significant (p < 0.05) increase in macrophage spreading as well as their phagocytic potential (i.e. phagocytic uptake and intracellular killing) of alveolar macrophages.

**Conclusions**

Clarithromycin has a great potential as an immunomodulatory antibiotic during *K. pneumoniae* B5055 induced pneumonia associated acute lung inflammation or injury (ALI).

## P9 Histone levels determined by an in-house immunoglobulin Y assay are associated to clinical outcomes in sepsis

### Jerico R. Santos^1^, Jesus Emmanuel A. D. Sevillejal^2,3^, Jose B. Nevado, Jr^2,4^

#### ^1^College of Medicine, University of the Philippines-Manila, Manila, Philippines; ^2^Institute of Molecular Biology and Biotechnology, National Institutes of Health, University of the Philippines-Manila, Manila, Philippines; ^3^Institute of Human Genetics, National Institutes of Health, University of the Philippines-Manila, Manila, Philippines; ^4^Department of Biochemistry and Molecular Biology, College of Medicine, University of the Philippines-Manila, Manila, Philippines

##### **Correspondence:** Jerico R. Santos (jerico.santos07@gmail.com)

**Background**

Experimental models have shown that histones are involved in the progression of sepsis and may be candidate prognostic biomarkers. There are limited prospective cohort studies of suspected septic patients aimed at determining the risk associated with increased level of histones to their progression to severe sepsis. To address this, we (1) developed an IgY-based immunoassay that can detect and quantify blood histone levels; (2) tested whether there is a difference in histone levels among septic and febrile patients that present in the emergency room; and (3) tested whether there is an association between histone levels in serum and the progression of patient to developing hypotension (sBP < 90 mmHg), azotemia, (creatinine > 2.0 mg/dL (176.8 μmol/L)) or thrombocytopenia (platelet count < 100,000/μL) using the developed IgY-based immunoassay.

**Materials and methods**

An indirect ELISA was developed for detecting human histones that used polyclonal immunoglobulin Y (IgY) extracted from eggs laid by calf histone-challenged chickens as a primary antibody. Chickens were inoculated with calf histones and the IgY from eggs were extracted by means of a delipidation, salt precipitation and affinity chromatography.

To determine its clinical utility as a prognostic kit, a prospective cohort study of 4 febrile patients and 21 septic patients seeking consult at the emergency room were enrolled based on the inclusion criteria. Higher total histone levels measured by immunoglobulin Y was then determined if it was related to the development of hypotension, azotemia or thrombocytopenia.

**Results**

**Fig. 8 (abstract P9).**
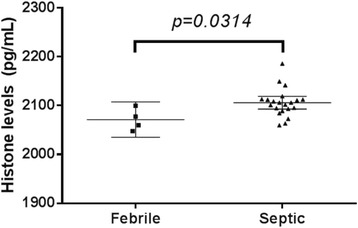
Histone Levels of Febrile and Septic Patients in the Emergency Room. Septic patients (Mean = 2106 pg/μL, 95 % CI, 2093–2119) have significantly higher (P < 0.05, two tailed t-test) levels of histones in their blood as compared to febrile patients (Mean = 2106 pg/μL, 95 % CI, 2104–2142)

The limit of detection of this IgY-based immunoassay was determined to be 2036 pg/mL. Comparison of histone levels between 4 febrile patients, and 21 septic patients within 3 days of admission in the hospital showed that septic patients have significantly higher (p = 0.0314) levels of histones as compared to febrile patients (Fig. 8).

**Fig. 9 (abstract P9).**
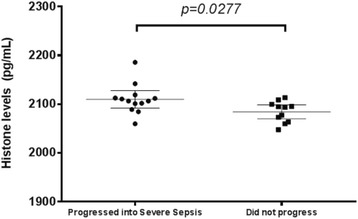
Patients that progressed into having hypotension, azotemia or thrombocytopenia have significantly higher histone levels than those that did not. In this cohort, those that progressed to severe sepsis (Mean = 2110, 95 % CI 2095–2125) have significantly higher levels of histones in their blood (P < 0.05, two-tailed t-test), as compared to those who did not progress (Mean = 2084, 95 % CI 2073–2096)

**Fig. 10 (abstract P9).**
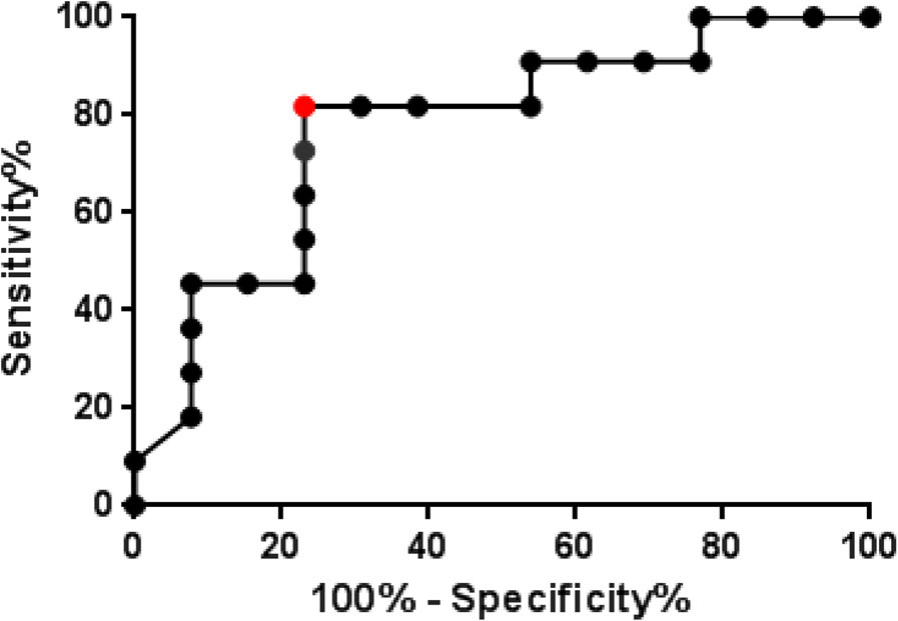
Receiver-Operating Characteristic (ROC) Curves for Prognosticating those that will progress into severe sepsis among the septic and febrile patients. The AUC is 0. 0.7727 (95 % CI, 0.5782 to 0.9672)

In this cohort, those that developed hypotension, azotemia or thrombocytopenia during their hospital stay have higher levels of histones upon admission (p = 0.0257) compared to those that did not (Fig. 9). Receiver operator curve analysis showed that at 2101 pg/uL (Fig. 10), likelihood ratio was 3.545 which had an estimated sensitivity (76.9 %), specificity (81.81 %), PPV (83.33), and NPV (75 %) (p = 012, Chi square test). At a cutoff of <2081 pg/uL, likelihood ratio was 5.909 of sensitivity (45.45 %) and specificity (92.31 %).

**Conclusions**

Histone levels may be predictive to the progression of defined clinical parameters related to worse outcomes in septic patients.

## P10 Inflammatory changes in renal tissues following Gram-positive pulmonary challenge

### Helena M. Linge, Kanta Ochani, Ke Lin, Ji Young Lee, Ping Wang, Manoj Tembhre, Shu Fang Liu, Pravin C. Singhal, Edmund J. Miller

#### Heart and Lung Research Unit, The Feinstein Institute for Medical Research, Northwell Health, Manhasset, NY USA

##### **Correspondence:** Edmund J. Miller (emiller@northwell.edu)

**Background**

Acute lung injury (ALI) is an important cause of morbidity and mortality in critically ill patients. Although the condition has many etiologies, pneumonia and sepsis are leading triggers. Age is a major determinant of clinical outcome, and there are age-dependent alterations in the responses to pulmonary challenge that can trigger kidney inflammation (a precursor of chronic kidney disease) and higher mortality rates. Staphylococcus aureus infection is a frequent cause of pneumonia, especially in elderly patients. To develop a better understanding of the pathophysiology, and assist in defining future targets, we developed a lung model of staphylococcal pneumonia in rodents. Using this model we have examined inflammatory responses, hemodynamics, and cardiac proteasome activation, neutrophil trafficking in pulmonary inflammation, and age-related changes [1–3]. Here we examined renal inflammation following pulmonary challenge.

**Materials and methods**

C57Bl6 Mice (8–10wks, n = 6-8/group) were challenged intratracheally with staphylococcal cell wall components, lipoteichoic acid (LTA), and peptidoglycan (PGN): (0.2 μg + 0.66 μg); (30 mg + 100 mg); (150 mg + 500 mg); or saline alone. Pulse oximetry was performed on awake animals before challenge and immediately prior to euthanasia. At 6, 24 or 72 hrs post challenge, animals were euthanized, bronchoalveolar lavage (BAL) performed, and tissues harvested. Concentrations of plasma and BAL cytokines were assessed by ELISA. The left kidney was fixed for histological analysis, and the right kidney was assessed for cytokine mRNA by qPCR, cytokine protein using ELISA, and activity of specific proteasome subunits by ProCISE [4].

**Results**

BAL from LTA/PGN challenged mice showed significant increases in neutrophils, total protein, IL-6, KC, MCP-1, TNFa and MIF as in previous studies. Additionally, while there was no LTA or PGN detected in the plasma, LTA/PGN challenged mice had increased plasma concentrations of IL-6, IL-1b, KC and TNFa. In renal tissue, there were time and concentration dependent changes in IL1b, IL-6, TNFa, IP10, and MCP-1 and an increase in proteasome b5 subunit activity.

**Conclusions**

Even in these young animals pulmonary challenge induced significant inflammatory changes within the lung, blood and kidney. Notably, in the kidney there was increased activity of proteasome b5, a regulator of IκB/NFκB activation and signaling pathways. Our recent study uncovered a time dependent, NF-kB switch that regulates transition from endothelial barrier injury to repair [5]. If we can define changes in the injury-to-repair phase in the kidney, we may reveal specific targets for therapeutic manipulation in a clinically relevant time frame to improve outcome from ALI associated renal involvement, particularly in the older individual.

**References**

1. Linge HM, Lee JY, Ochani K, Koga K, Kohn N, Ojamaa K, Powell SR, Miller EJ: Age influences inflammatory responses, hemodynamics, and cardiac proteasome activation during acute lung injury. Exp Lung Res 2015, 41(4):216–227.

2. Palestro C, Linge HM, Nichols KJ, Ochani K, Bhargava KK, Miller EJ: Neutrophil Trafficking in Pulmonary Inflammation: Monitoring Migration and Blockade with 111In-Labeled Leukocytes Journal of Pulmonary & Respiratory Medicine 2015, 5:289–297.

3. Lee JY, Linge HM, Ochani K, Lin K, Miller EJ: N-Ethylmaleimide Sensitive Factor (NSF) Inhibition Prevents Vascular Instability following Gram-Positive Pulmonary Challenge. PLoS One 2016, 11(6):e0157837.

4. Kirk CJ, Powell SR, Miller EJ: Assessment of cytokine-modulated proteasome activity. Methods Mol Biol 2014, 1172:147–162.

5. Liu G, Ye X, Miller EJ, Liu SF: NF-kappaB-to-AP-1 switch: a mechanism regulating transition from endothelial barrier injury to repair in endotoxemic mice. Sci Rep 2014, 4:5543.

## P11 Cathelicidin protects against intestinal barrier dysfunction in polymicrobial sepsis

### Jeffery HO^1^, Xiaodong Liu^1^, Thomas Kwong^2^, Lin Zhang^1^, Hung Chan^1^, Sunny H Wong^2^, Gordon Choi^1^, Tony Gin^1^, Matthew TV Chan^1^, William KK Wu^1^

#### ^1^Department of Anaesthesia and Intensive Care, Prince of Wales Hospital, The Chinese University of Hong Kong, Shatin, Hong Kong; ^2^Department of Medicine and Therapeutics, Prince of Wales Hospital, The Chinese University of Hong Kong, Shatin, Hong Kong

##### **Correspondence:** Jeffery HO (jeffho@cuhk.edu.hk)

**Background**

Accumulating evidence suggests that the intestinal barrier function is impaired during systemic inflammation as in sepsis. Animal models of sepsis revealed several ileal pathological changes. These include epithelial apoptosis, disruption of tight junctions and increased intestinal permeability [1,2]. The impaired gut barrier function may increase the risk of bacterial translocation from the gut lumen to the bloodstream, aggravating systemic inflammation. The molecular mechanism associated with this phenotype remains largely unknown. Cathelicidin represents one of the most important classes of antimicrobial peptides in mammals. In addition to bactericidal property, this peptide inhibits endotoxin-induced pyroptosis of leukocytes, suppresses the release of inflammatory mediators and protects endothelial cells from apoptosis [3–5]. In this study, we aimed to investigate the role of murine cathelicidin-related antimicrobial peptide (mCRAM), a rodent antimicrobial peptide analogous to human LL-37, in maintaining gut barrier function in sepsis.

**Materials and methods**

129X1/SvJ mice that were wild type (cnlp+/+) or deficient for cathelicidin (cnlp−/−) were used. Polymicrobial sepsis was induced by cecal-ligation and puncture (CLP) [6]. Animals were divided into four groups: cathelicidin wild type and knockout mice receiving either CLP or sham surgery. At 24 h, blood and ileal tissues were harvested for total bacterial DNA quantification, immunoblotting and histological staining. The survival rates and septic severity were recorded every 12 hours until seven days after the surgery. Sepsis morbidity was evaluated by Murine Sepsis Severity (MSS) score [7]. At 20 h after CLP or sham surgery, the animals were orally fed with 4 kDa fluorescein-dextran. Three hours later, serum was harvested and fluorescence was measured.

**Results**

**Fig. 11 (abstract P11).**
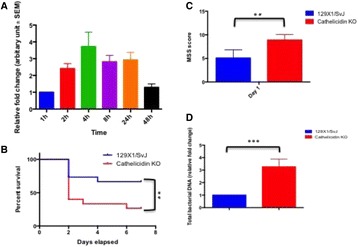
Cathelicidin is upregulated after cecal-ligation and puncture induced sepsis in 129X1/SvJ (cnlp+/+) mice from which total RNA and protein were collected from distal ileums over a period of time for (**a**) real time PCR targeting clnp for mCRAMP. Genetic knockout of cnlp led to (**b**) reduced survival, (**c**) higher sepsis severity score and (**d**) increased bacterial DNAemia upon experimental sepsis. Error bars represents standard error of the mean. P < 0.05 denotes statistical significance, Mann–Whitney U-test

Knocking out cnlp gene has significantly increased septic severity and reduced 7-day survival of 129X1/SvJ mice. Real time PCR targeting bacterial 16S rDNA revealed that total bacterial DNA load increased more than threefold (Fig. 11).

**Fig. 12 (abstract P11).**
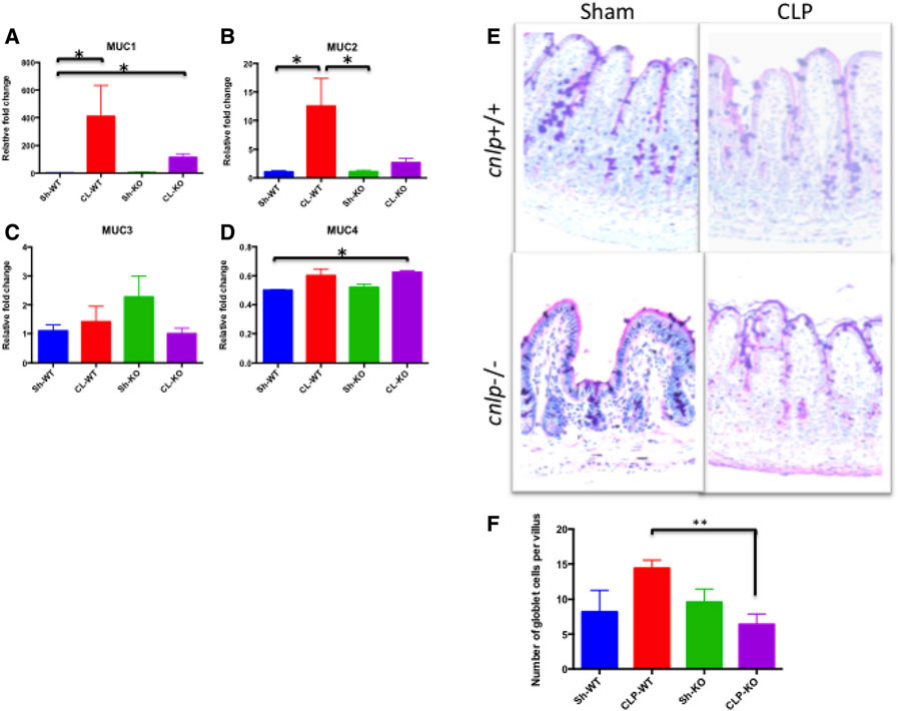
Effects of cecal-ligation and puncture (CL) or sham surgery (Sh) on (**a**-**d**) the expression of mucin genes MUC1-4 and (**e**) acid mucin in distal ileum of cathelicidin wildtype (WT) or knockout (KO) mice at 24 h after CL as determined by quantitative real-time PCR and Alcian blue perioidic acid Schiff reaction, respectively. (**f**) The number of acid-mucin producing globlet cells per villus was compared. Error bars represent standard error of the mean

**Fig. 13 (abstract P11).**
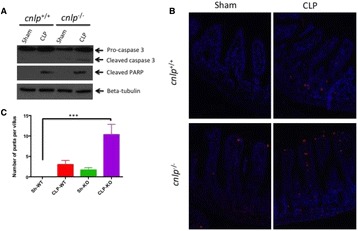
Increased apoptosis was detected in cathelicidin-knockout (cnlp−/−) mice compared to -wild type mice (cnlp+/+) after CL-induced sepsis as demonstrated by (**a**) immunoblotting for cleaved caspase-3 and poly ADP ribose polymerase (PARP), and (**b**) TUNEL staining. Quantification was shown, for TUNEL, as (**c**) number of punta per villus. All specimens were collected at 24 hr after CL or Sham surgery. Error bars denote standard error of the mean. P < 0.05 denotes statistical significance, Kruskal-Wallis test

**Fig. 14 (abstract P11).**
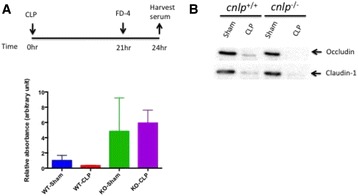
Deletion of cnlp increased ileal permeability as evidenced by (**a**) increased serum concentration of FITC-labeled dextran 4kD (FD-4) at 3 hr after oral garvage following CL. (**b**) The protein level of occludin and claudin-1 was determined by immunoblotting. Error bars denote standard error of the mean

Without cathelicidin, the expression of mucus has also reduced considerably, as revealed by Alcian blue periodic acid Schiff reaction and RT-PCR targeting MUC1, MUC2, MUC3 and MUC4 genes (Fig. 12). Immunoblotting revealed cleavage of caspase 3 and PARP involving in apoptosis (Fig. 13). Consistently, confocal microscopy using TUNEL staining identified greater number of puncta per villus. Deletion of cnlp gene increased ileal permeability to 4kD Fluorescein-labelled dextran, accompanied with reduced expression of tight junction proteins claudin-1 and occludin (Fig. 14).

**Conclusions**

This study demonstrated the critical role of cathelicidin in maintaining gut barrier integrity in polymicrobial sepsis. This may partially explain the association between lower LL-37 and higher mortality in septic patients [8].

**References**

[1] Li Q, Zhang Q, Wang C et al. Disruption of tight junctions during polymicrobial sepsis in vivo. J Pathol. 2008; 218: 210–221.

[2] Wu W, Jiang RL, Wang JC et al. Effect of Shenfu injection on intestinal mucosal barrier in a rat model of sepsis. Am J Emerg Med. 2015; 33: 1237–1243.

[3] Hu Z, Murakami T, Suzuki K et al. Antimicrobial cathelicidin peptide LL-37 inhibits the LPS-ATP-induced pyroptosis of macrophages by dual mechanism. PLoS One. 2014; 9:e85765.

[4] Murakami T, Obata T, Kuwahara-Arai K et al. Human antimicrobial cathelicidin peptide LL-37 suppresses the production and release of septic mediators in D-galactosamine-sensitized endotoxin shock mice. Int Immunol. 2009; 21:905–912.

[5] Suzuki K, Murakami T, Kuwahara-Arai K et al. Human antimicrobial cathelicidin peptide LL-37 suppresses the LPD-induced apoptosis of endothelial cells. Int Immunol. 2011; 23:185–93.

[6] Rittirsch D, Huber-Lang MS, Flierl MA et al. Immunodesign of experimental sepsis by cecal ligation and puncture. Nat Prot. 2009; 4: 31–36.

[7] Shrum B, Anantha RV, Xu SX et al. A robust scoring system to evaluate sepsis severity in an animal model. BMC Res Notes. 2014; 7: 233.

[8] Leaf DE, Croy HE, Abrahams SJ et al. Cathelicidin antimicrobial protein, vitamin D, and risk of death in critically ill patients. Crit Care 2015; 19:80.

## P12

**Withdrawn**

## P13 Proline-specific peptidases in the plasma of patients with septic shock: their potential as biomarker and associations with established parameters

### Gwendolyn Vliegen^1^, Kaat Kehoe^1^, Robert Verkerk^1^, Erik Fransen^2^, Esther Peters^3,4^, Anne-Marie Lambeir^1^, Peter Pickkers^3^, Philippe G. Jorens^5^, Ingrid De Meester^1^

#### ^1^ Laboratory of Medical Biochemistry, University of Antwerp, Universiteitsplein 1, 2610 Wilrijk, Belgium; ^2^ StatUa Center for Statistics, University of Antwerp, Prins Boudewijnlaan 43, 2650 Edegem, Belgium; ^3^ Department of Intensive Care Medicine, Radboud university medical center, 6500HB Nijmegen, The Netherlands; ^4^ Department of Pharmacology and Toxicology, Radboud university medical center, 6500HB Nijmegen, The Netherlands; ^5^ Department of Critical Care Medicine, Antwerp University Hospital and Laboratory of Experimental Medicine and Pediatrics, University of Antwerp, Wilrijkstraat 10, 2650 Edegem, Belgium

##### **Correspondence:** Gwendolyn Vliegen (gwendolyn.vliegen@uantwerpen.be)

**Background**

To date, there is no standard diagnostic test to identify septic patients, although early identification is necessary to improve their outcome [1]. We sought to identify novel biomarkers by studying the enzymatic activity of the proline-specific peptidases dipeptidyl peptidase 4 (DPP4), prolyl carboxypeptidase (PRCP), fibroblast activation protein α (FAP) and prolyl oligopeptidase (PREP) in the plasma of 32 controls and 40 patients with septic shock on days 1, 3, 5 and 7.

**Materials and methods**

Enzymatic activity measurements were done using in-house developed specific assays [2–4]. On the same days, inflammatory (e.g. TNF-α, IL-6) , hemodynamic (e.g. mean arterial pressure, pulse) and metabolic parameters (e.g. bilirubin, lactate) were measured as well. The data was used to generate receiver operating characteristic (ROC) curves and to identify associations. A survival analysis (up to 90 days) was also performed.

**Results**

Significant differences between the control group and the patients with septic shock could be found for all four enzymes. PREP and PRCP showed a higher enzymatic activity on day 1 compared to the controls (p ≤ 0.05 and p ≤ 0.01, respectively). FAP and DPP4, on the other hand, were both much lower in the patients group on all studied time points (p ≤ 0.001). PREP, FAP and DPP4 showed to be very good in discriminating cases from controls with area under the curve (AUC) values of 0.91 (CI: 0.85-0.99), 0.96 (CI: 0.92-1) and 0.94 (CI: 0.89-0.99), respectively. PRCP had a lower predicting value with an AUC of 0.69 (CI: 0.58-0.82). A nominally significant association was observed between survival and the DPP4 enzymatic activity at day 1 (p ≤ 0.05), with a higher DPP4 enzymatic activity being associated with an increase in survival. Several interesting positive associations, such as PRCP with lactate, PREP with IL-1 receptor antagonist and PREP with intestinal fatty-acid binding protein, were identified. PRCP and PREP were also associated with the dosage of noradrenalin administered to maintain an acceptable mean arterial pressure.

**Conclusions**

DPP4, FAP and PREP showed to be good in discriminating between controls and cases and should be further explored as possible candidate biomarkers for sepsis. The associations found also warrant further research.

1. Dellinger RP, Levy MM, Rhodes A, Annane D, Gerlach H, Opal SM, et al. Surviving Sepsis Campaign. Crit. Care Med. 2013;41:580–637.

2. Matheeussen V, Lambeir A-M, Jungraithmayr W, Gomez N, Mc Entee K, Van der Veken P, et al. Method comparison of dipeptidyl peptidase IV activity assays and their application in biological samples containing reversible inhibitors. Clin. Chim. Acta. 2012;413:456–62.

3. Kehoe K, Verkerk R, Sim Y, Waumans Y, Van der Veken P, Lambeir A-M, et al. Validation of a specific prolylcarboxypeptidase activity assay and its suitability for plasma and serum measurements. Anal. Biochem. 2013;443:232–9.

4. Goossens F, De Meester I, Vanhoof G, Scharpé S. A sensitive method for the assay of serum prolyl endopeptidase. Eur. J. Clin. Chem. Clin. Biochem. 1992;30:235–8.

## P14

**Withdrawn**

## P15 Dexamethasone prevents epithelial barrier dysfunction in endotoxemic rats

### Aline Barbosa Ribeiro, Ana Paula Trevelin Souza, Humberto Giusti, Celso Rodrigues Franci, Rafael Simone Saia

#### Department of Physiology, Ribeirão Preto Medical School, University of São Paulo, Ribeirão Preto, São Paulo, Brazil

##### **Correspondence:** Rafael Simone Saia (rssaia@fmrp.usp.br)

**Background**

The systemic inflammation represents a primary injury to the intestinal epithelium structure. In acute phase, the massive production of inflammatory mediators (cytokines and nitric oxide) contributes to the intestinal permeability, bacterial translocation and tight junction rearrangement. During the past decades, the clinical administration of glucocorticoids has been widely discussed and the Surviving Sepsis Campaign’s recommendation is the low-dose treatment for those patients refractory to vasopressor therapy. Our aim was to investigate the possible role of dexamethasone on lipopolysaccharide (LPS)-induced intestinal epithelial barrier dysfunction. Furthermore, we evaluated its ability to modulate the inflammatory response and also the expression of proteins of the intestinal tight junctions.

**Materials and methods**

To conduct these experiments, male rats had their jugular vein cannulated for endotoxin administration, one day before the experiment. Rats were pre-treated with dexamethasone (synthetic glucocorticoid; 0.1 or 1 mg/kg, intraperitoneal) before LPS administration (1.5 mg/kg, intravenous). At 6 h after endotoxemia induction, the intestinal permeability was evaluated by injecting FITC-dextran 4 kDa in the ileum, mesenteric lymph nodes were collected for microbiological analysis and also cytokines were quantified in the plasma and intestinal mucosa by ELISA technique. Additionally, the integrity of the tight junctions was determined by transmission electron microscopy, histological analysis, the expression of tight junction proteins (occludin, claudin-1, claudin-2, junctional adhesion molecule-A) as well as their localization.

**Results**

Our results demonstrated that dexamethasone administration reduces the LPS-induced permeability in the ileum and prevented the bacterial translocation to the mesenteric lymph nodes. The plasma and mucosa concentrations of TNF-α, IL-1β, IL-6, IL-10 and IFN-γ were significantly reduced in dexamethasone-treated rats. Furthermore, treatment with dexamethasone reverted the LPS-induced epithelial barrier dysfunction, increasing the expression of occludin and claudin-1, reducing the claudin-2 cleavage and also the localization of these proteins at the apical portion. Moreover, the histological damages and the morphology of the tight junctions were preserved by the dexamethasone administration, therefore reducing their opening induced by endotoxemia.

**Conclusions**

Together these results suggest a protective role for dexamethasone preventing the intestinal barrier dysfunction induced by systemic inflammation, possibly modulating the inflammatory response. Furthermore, these experimental findings reinforce the Surviving Sepsis Campaign’s recommendation for the administration of low glucocorticoid doses in septic shock patients.

**Acknowledgements**

CNPq and FAPESP

## P16 Cytokine response in adult severe sepsis patients with features of macrophage activation syndrome

### Renee R. Anderko^1^, Vanessa M. Jackson^1^, Octavia M. Peck Palmer^1,2^, Derek C. Angus^1^, John A. Kellum^1,3^, Joseph A. Carcillo^1,4^

#### ^1^Department of Critical Care Medicine, University of Pittsburgh School of Medicine, Pittsburgh, PA, USA; ^2^University of Pittsburgh Medical Center, Pittsburgh, PA, USA; Department of Pathology, University of Pittsburgh School of Medicine, Pittsburgh, PA, USA; ^3^Center for Critical Care Nephrology, University of Pittsburgh, Pittsburgh, PA, USA; ^4^Department of Pediatrics, University of Pittsburgh School of Medicine, Pittsburgh, PA, USA; Children’s Hospital of Pittsburgh of University of Pittsburgh Medical Center, Pittsburgh, PA, USA

##### **Correspondence:** Renee R. Anderko (anderkorr@upmc.edu)

**Background**

Shakoory and colleagues performed a post hoc analysis of a randomized clinical trial of anakinra, an interleukin-1 receptor antagonist [1], and reported that severe sepsis patients with features of macrophage activation syndrome (MAS), defined by the combined presence of hepatobiliary dysfunction and disseminated intravascular coagulation, represented 5.6 % of the severe sepsis population. Additionally, anakinra, in comparison with placebo, reduced 28-day mortality (34.6 % vs 64.7 %; p < 0.05) in a subset of patients with features of MAS [1]. Based on these findings, we hypothesized that the Protocolized Care for Early Septic Shock (ProCESS) trial [2] would contain a similar proportion of septic patients with features of MAS and a similar mortality rate. We further hypothesized that MAS patients would have elevated circulating biomarkers related to macrophage activation, including ferritin, interleukin-18 (IL-18), interleukin-1β (IL-1β), and interferon-γ (IFN-γ).

**Materials and methods**

We selected all patients with features of MAS and age-matched controls without features of MAS from the ProCESS trial. Concentrations of circulating ferritin, IL-18, IL-1β, and IFN-γ were measured in plasma samples collected on day 1, and mortality was assessed at hospital discharge and at 90 days. The MAS patients were further stratified according to their circulating ferritin levels, and a threshold of 1200 ng/ml was established based on the upper limit of the 95 % confidence interval for the mean ferritin concentration in MAS survivors.

**Results**

Of the 1341 ProCESS subjects, 82 exhibited features of MAS (6.1 %). In-hospital and 90-day mortality rates were higher in the MAS patients than the non-MAS patients (42.7 % vs 9.8 % and 61.0 % vs 28.1 %; p < 0.05). As outlined in Table 3, MAS subjects showed higher circulating ferritin, IL-18, and IL-1β concentrations (p < 0.05). Approximately 27 % of septic patients with features of MAS had ferritin concentrations greater than 1200 ng/ml. Interestingly, these patients had higher in-hospital mortality (63.6 %) and 90-day mortality (72.7 %), as well as higher IL-18, IL-1β, and IFN-γ concentrations compared to the septic patients without MAS (p < 0.05) (Table 4).

**Conclusions**

Post hoc analysis of ProCESS patients illustrated that features of MAS are relatively uncommon but lethal in severe sepsis. Septic patients with features of MAS and ferritin levels greater than 1200 ng/ml had the highest mortality and increased IL-18, IL-1β, and IFN-γ concentrations compared to non-MAS septic patients, making this an attractive subset to target in future anti-inflammatory agent trials.

**References**

1. Shakoory B, Carcillo JA, Chatham WW, Amdur RL, Zhao H, Dinarello CA, Cron RQ, Opal SM: Interleukin-1 receptor blockade is associated with reduced mortality in sepsis patients with features of macrophage activation syndrome: reanalysis of a prior phase III trial. Critical Care Medicine 2016, 44:275–281.

2. ProCESS Investigators, Yealy DM, Kellum JA, Huang DT, Barnato AE, Weissfeld LA, Pike F, Terndrup T, Wang HE, Hou PC, et al.: A randomized trial of protocol-based care for early septic shock. The New England Journal of Medicine 2014, 370:1683–1693.

**Table 3 (abstract P16).** Clinical characteristics of septic patients with and without features of MAS

Biochemical Profile			
	Septic Patients with MAS (n = 82)	Septic Patients without MAS (n = 82)	P value*
Ferritin, ng/ml, 0 hr mean (sd, median, range) [95 % CI]	1834.49 (3316.41, 482.00, 14.30-17,502.00) [1095.30, 2573.70]	444.65 (846.45, 213.90, 5.70-6536.00) [257.17, 632.12]	<0.0001
IL-18, pg/ml, 0 hr mean (sd, median, range) [95 % CI]	1582.68 (2273.23, 847.04, 123.61-15,214.01) [1079.20, 2086.20]	558.13 (499.23, 408.62, 159.84-3107.64) [446.86, 669.41]	<0.0001
IFN-γ, pg/ml, 0 hr mean (sd, median, range) [95 % CI]	115.29 (534.76, 12.28, 0.10-4704.60) [−3.15, 233.73]	27.62 (71.38, 0.10, 0.10-590.33) [11.71, 43.53]	0.1413
IL-1β, pg/ml, 0 hr mean (sd, median, range) [95 % CI]	69.21 (176.85, 8.92, 0.08-928.70) [26.99, 111.42]	12.96 (29.25, 4.74, 0.08-183.43) [5.53, 20.38]	0.0001
Mortality			
In-hospital mortality, %	42.7	9.8	<0.0001
90-day mortality, %	61.0	28.1	<0.0001

*The p values represent a comparison between the patients with features of macrophage activation syndrome (MAS) and the patients without features of MAS. We used Fisher’s Exact test for categorical measures and the Mann–Whitney U test for continuous and non-normally distributed measures

**Table 4 (abstract P16).** Clinical characteristics of septic patients with features of MAS and ferritin concentrations >1200 ng/ml vs septic patients without features of MAS

Biochemical Profile			
	Septic Patients without MAS (n = 82)	Septic Patients with MAS and Ferritin >1200 (n = 22)	P value*
Ferritin, ng/ml, 0 hr mean (sd, median, range) [95 % CI]	444.65 (846.45, 213.90, 5.70-6536.00) [257.17, 632.12]	5584.60 (4561.20, 3535.00, 1345.30-17,502.00) [3561.90, 7607.20]	<0.0001
IL-18, pg/ml, 0 hr mean (sd, median, range) [95 % CI]	558.13 (499.23, 408.62, 159.84-3107.64) [446.86, 669.41]	2625.30 (3513.40, 1252.40, 305.85-15,214.01) [1067.30, 4183.40]	<0.0001
IFN-γ, pg/ml, 0 hr mean (sd, median, range) [95 % CI]	27.62 (71.38, 0.10, 0.10-590.33) [11.71, 43.53]	287.61 (997.26, 34.09, 0.10-4704.60) [−154.63, 729.85]	0.0287
IL-1β, pg/ml, 0 hr mean (sd, median, range) [95 % CI]	12.96 (29.25, 4.74, 0.08-183.43) [5.53, 20.38]	45.32 (94.76, 8.55, 0.08-395.15) [2.19, 88.46]	0.0074
Mortality			
In-hospital mortality, %	9.8	63.6	<0.0001
90-day mortality, %	28.1	72.7	0.0003

*The p values represent a comparison between the patients without features of MAS and the patients with features of MAS and ferritin concentrations >1200 ng/ml. We used Fisher’s Exact test for categorical measures and the Mann–Whitney U test for continuous and non-normally distributed measures

## P17 Evaluation of a novel molecular host response assay to diagnose infection in patients after high-risk gastro-intestinal surgery: a pilot study

### D.M. Verboom^1,2^, M.E. Koster-Brouwer^1,2^, K. van de Groep^1,2^, J.F. Frencken^1,2^, B. Scicluna^3^, S.S. Gisbertz^4^, M.I. van Berge Henegouwen^4^, J.P. Ruurda^5^, R. van Hillegersberg^5^, T. van der Poll^3,6,7^, M.J.M. Bonten^1,8^, O.L. Cremer^2^

#### ^1^Julius Center for health sciences and primary care, University Medical Center Utrecht, Utrecht, the Netherlands; ^2^Department of Intensive Care, University Medical Center Utrecht, Utrecht, the Netherlands; ^3^Center for Experimental and Molecular Medicine, Academic Medical Center, University of Amsterdam, Amsterdam, the Netherlands; ^4^Department of Surgery, Academic Medical Center, Amsterdam, the Netherlands; ^5^Department of Surgery, University Medical Center Utrecht, Utrecht, the Netherlands.; ^6^Center for Infection and Immunity, Academic Medical Center, Amsterdam, the Netherlands; ^7^Division of Infectious Diseases, Academic Medical Center, Amsterdam, the Netherlands|; ^8^Department of Medical Microbiology, University Medical Center Utrecht, Utrecht, the Netherlands

##### **Correspondence:** D.M. Verboom (D.M.Verboom@umcutrecht.nl)

**Conflicts of interest**

The authors declare that they do not have a conflict of interest related to the subject matter.

**Background**

Patients after esophagectomy are prone to develop sepsis due to pneumonia, anastomotic leakage, or wound infection. A new diagnostic test (SeptiCyte LAB) that measures the expression of four blood gene biomarkers has diagnostic utility in distinguishing sepsis from SIRS [1]. However, test performance in the immediate postoperative setting has not been investigated.

**Materials and methods**

We studied patients undergoing esophagectomy in two Dutch university hospitals from 2011 to 2013 who had developed a complication requiring ICU (re)admission within 30 days. Blood was collected postoperatively and when a complication occurred. After RNA extraction, samples were analyzed on an Applied Biosystems® 7500 fast Dx Real-Time PCR instrument. SeptiCyte LAB results (a score, range 0–10) were categorized into four probability bands according to the manufacturer's specification.

**Results**

Among 384 patients 113 (29 %) subjects had developed at least one complication and were thus eligible for study inclusion; paired samples (i.e., both a direct postoperative and follow-up sample) were available for 71 (63 %) of these. Sample preparation or processing issues resulted in exclusion of 4 pairs, leaving 67 (59 %) patients for analysis (Table 5).

**Fig. 15 (abstract P17).**
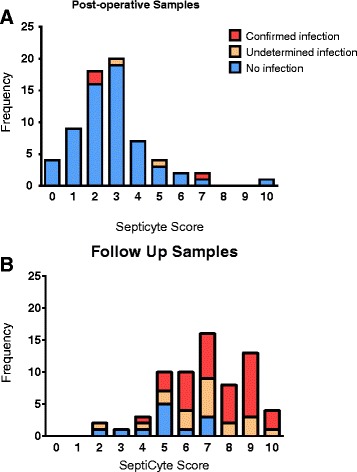
Distribution of SeptiCyte LAB scores in samples taken immediately after surgery and at onset of a postoperative complication

Five (7 %) patients had active infection during postoperative sampling, and 55 (82 %) on the day of their first complication. SeptiCyte results were highly variable immediately after surgery and at follow-up (Fig. 15), but scores increased significantly as complications evolved (median 2.7 (IQR 1.6-3.4) versus 7.3 (5.7-8.7); p < 0.001). Importantly, samples taken during infection yielded higher scores then specimens taken at onset of a non-infectious complication (median 7.3 (5.7-8.7) versus 5.2 (4–6.3); p < 0.001).

Considering all 134 samples separately, 45 were classified as 'sepsis not likely' based on a SeptiCyte score <3.1; among these, we observed 2 cases of confirmed infection (false negative rate 4 %). Likewise, among 89 events with a score >3.1, infection was ruled-out or never clinically suspected in 33 cases (false positive rate 37 %). The incorporation of SeptiCyte LAB results markedly improved the performance of SIRS criteria in classifying confirmed infections (c-statistic 0.86 (95%CI 0.80-0.93) versus 0.97 (95%CI 0.94-0.99); p < 0.001). However, a final diagnosis of infection could not be made with certainty on 21 occasions (including two postoperative samples), which precluded formal estimation of test characteristics.

**Conclusion**

SeptiCyte LAB is a novel sepsis biomarker which may have clinical utility in monitoring patients at high risk for postoperative infections, but its diagnostic value in this setting has to be further evaluated.

**Acknowledgements**

We thank ImmunExpress for kindly providing lab kits and technical assistance.

**References**

1. McHugh L, Seldon TA, Brandon RA, Kirk JT, Rapisarda A, Sutherland AJ, Presneill JJ, Venter DJ, Lipman J, Thomas MR, et al. A Molecular Host Response Assay to Discriminate Between Sepsis and Infection-Negative Systemic Inflammation in Critically Ill Patients: Discovery and Validation in Independent Cohorts. PLoS Med, 2015. **12**(12): p. e1001916.

2. Klein Klouwenberg PM, Ong DS, Bos LD, de Beer FM, van Hooijdonk RT, Huson MA, Straat M, van Vught LA, Wieske L, Horn J, et al., Interobserver agreement of Centers for Disease Control and Prevention criteria for classifying infections in critically ill patients. Crit Care Med, 2013. **41**(10): p. 2373–2378.

**Table 5 (abstract P17).** Patient characteristics by infection status at the time of a first postoperative complication (n = 67)

	Post-hoc likelihood of infection			*P*-Value
	No infection*	Undetermined	Confirmed infection	
	(N = 12)	(N = 19)	(N = 36)	
Age, years	65.5 (61.0, 72.0)	67.0 (53.0, 73.0)	65.0 (59.0, 69.0)	0.90
Charlson Comorbidity Index	5.5 (0.0, 6.9)	6.4 (0.0, 6.4)	6.4 (5.1, 9.5)	0.15
SOFA score	5.0 (2.0, 6.0)	5.0 (3.0, 6.0)	6.0 (4.0, 8.0)	0.24
Complication onset, days after surgery	3.0 (2.0, 4.0)	5.0 (3.0, 11.0)	6.5 (3.0, 8.0)	0.09
ICU length of stay following onset complication, days	1.5 (1.0, 5.0)	2.0 (1.0, 4.0)	8.5 (4.5, 16.5)	<0.001
Hospital mortality	1 (8 %)	1 (5 %)	12 (33 %)	0.03
*SIRS criteria*				
T > 38 °C or T < 36 °C	4 (33 %)	7 (37 %)	13 (36 %)	0.98
WBC <4 or >12 (10^9^/ml)	4 (33 %)	10 (53 %)	23 (64 %)	0.18
Respiratory rate >20/min, PCO2 < 32 mmHg or mechanical ventilation	5 (42 %)	11 (58 %)	31 (86 %)	0.01
Heart rate >90/min	6 (50 %)	13 (68 %)	28 (78 %)	0.19

The likelihood of infection was based on post hoc physician assessment using validated definitions [2]. Patients considered to have 'possible' infection were classified as undetermined; patients considered to have 'probable' or 'definite' infection were classified as confirmed. *These include 5 patients initially treated with antibiotics in whom infection was later ruled out, and 7 patients in whom infection was never clinically suspected

Data show medians (Q1 – Q3) or n (%). SOFA = Sequential Organ Failure Assessment, SIRS = Systemic Inflammatory Response Syndrome, T = Temperature, WBC = White blood cell count

## P18 Validation of sepsis-3 diagnostic criteria in intensive care unit patients

### D.M. Verboom^1,2^, J.F. Frencken^1,2^, T. van der Poll^3,4^, M.J.M. Bonten^1,5^, O.L. Cremer^2^, P.M.C. Klein Klouwenberg^5^

#### ^1^ Julius Center for Health Sciences and Primary Care, University Medical Center Utrecht, Utrecht, the Netherlands; ^2^ Department of Intensive Care, University Medical Center Utrecht, Utrecht, the Netherlands; ^3^ Center for Infection and Immunity, Academic Medical Center, Amsterdam, the Netherlands; ^4^ Division of Infectious Diseases, Academic Medical Center, Amsterdam, the Netherlands; ^5^ Department of Medical Microbiology, University Medical Center Utrecht, Utrecht, the Netherlands

##### **Correspondence:** D.M. Verboom (D.M.Verboom@umcutrecht.nl)

**Conflicts of interest**

The authors declare that they do not have a conflict of interest related to the subject matter.

**Background**

The Third International Consensus definitions for sepsis aimed to improve the discrimination between sepsis and uncomplicated infections [1]. Our goal was to assess the construct and criterion validity of the new definitions in a cohort of ICU patients with infection.

**Methods**

We analyzed ICU patients with suspected infection who were enrolled as part of a prospective cohort study in the Netherlands between June 2011 and April 2015 [2]. Infections were classified as either present at admission or newly-acquired in the ICU (onset >48 hours). Sepsis and septic shock were defined according to both old and updated criteria [1,3]. The apparent incidences of sepsis and septic shock according to both definitions, and associated hospital mortality rates were compared. In addition, we explored minor variations in the timing of diagnostic criteria relative to infection onset, and inclusion of lactate for the definition of septic shock. Sepsis status was added to a baseline logistic model (including age, gender, race and chronic comorbidity) to evaluate the value of the sepsis-3 criteria in predicting mortality.

**Results**

Among 1582 included patients, 1081 had suspected infection at admission; 1020 (94 %) and 391 (36 %) of these fulfilled sepsis and shock criteria according to the sepsis-3 definitions, compared to 1044 (97 %) and 290 (27 %) for the old definitions (agreement 92 % and 81 %, respectively). Mortality was 28 % (95%CI 25–31) in patients meeting sepsis-3 criteria compared to 27 % (95%CI 25–30) for new definitions, this was 42 % (95%CI 37–47) and 50 % (95%CI 45–56) for septic shock (Figure 1). Among 501 patients with ICU-acquired infection, 298 (59 %) and 141 (28 %) patients as compared to 497 (98 %) and 96 (19 %) patients fulfilled the new and old definitions, respectively (agreement 60 % and 73 %). Mortality in sepsis was 38 % (95%CI 33–44) and 35 % (95%CI 31–39) and was 50 % (95%CI 41–58) versus 69 % (95%CI 59–78) for septic shock. Although the observed incidence of sepsis and septic shock varied widely by both variations in the time window used for defining sepsis and inclusion of lactate for defining shock, neither modification impacted mortality (Figure 1). Sepsis-3 had small, but statistically significant incremental value in predicting mortality in patients with infection at admission (AUC 0.62 versus 0.60;p = 0.0002), nearly equal the old sepsis definition in this respect (AUC 0.61 versus 0.60;p = 0.0338).

**Conclusion**

Agreement between new and old sepsis definitions was higher in infections at admission than in ICU-acquired infections. Mortality was similar using the various sepsis definitions. Sepsis-3 had no clinical relevant additive value in predicting mortality infections at admission.

**References**

1. Singer M, Deutschman CS, Seymour CW, Shankar-Hari M, Annane D, Bauer M, Bellomo R, Bernard GR, Chiche JD, Coopersmith CM *et al.*: The Third International Consensus Definitions for Sepsis and Septic Shock (Sepsis-3). *Jama* 2016, 315(8):801–810.

2. Klein Klouwenberg PM, Ong DS, Bos LD, de Beer FM, van Hooijdonk RT, Huson MA, Straat M, van Vught LA, Wieske L, Horn J *et al.*: Interobserver agreement of Centers for Disease Control and Prevention criteria for classifying infections in critically ill patients. *Critical care medicine* 2013, 41(10):2373–2378.

3. Bone RC, Balk RA, Cerra FB, Dellinger RP, Fein AM, Knaus WA, Schein RM, Sibbald WJ: Definitions for sepsis and organ failure and guidelines for the use of innovative therapies in sepsis. The ACCP/SCCM Consensus Conference Committee. American College of Chest Physicians/Society of Critical Care Medicine. *Chest* 1992, 101(6):1644–1655.

## P19 Aromatic metabolites clearance reflects the efficiency of ICU treatment more accurately then lactate clearance

### Natalia Beloborodova, Artem Osipov, Alisa Pautova, Aleksandra Bedova

#### Negovsky Research Institute of General Reanimatology, Moscow, Russia

##### **Correspondence:** Natalia Beloborodova (nvbeloborodova@yandex.ru)

**Introduction**

Metabolites of aromatic amino acids - phenylcarboxylic acids (PhCAs) - significantly increased in critically ill patients with infection, especially with sepsis [1] and associated with mortality [2, 3]. They have demonstrated good predictive ability in patients with different critical illness [3, 4]. Unfortunately, accurate prediction does not save the patient's life. We need better criteria for evaluating of treatment efficiency in patients with infection. This work is our first step in the development of laboratory monitoring based on PhCA clearance.

**Methods**

High-risk patients with median age 60 (IR 53–74) years with acute abdomen (n = 45, male 51 %) were admitted to the ICU immediately after emergency surgery. Acute abdomen development was associated with intestinal perforation (n = 26) and/or acute intestinal obstruction (n = 19). Serum samples were collected on the first and the third days of treatment in the ICU. The total level (C) of three PhCAs (phenyllactic, p-hydroxyphenyllactic and p-hydroxyphenylacetic acids) was measured using previously described gas chromatography methodology [5]. Lactate concentration (C) and SOFA scores were registered. PhCA and lactate clearance was calculated as followed: [(C_first_-C_third_)/C_first_]x100%. The efficiency of treatment was judged based on the ICU outcome. Statistical processing was performed using IBM SPSS Statistics 22 program.

**Results**

The results were divided into two sub-groups and were presented as medians with 25-75 % interquartile range depending on the outcome (Table 6).

**Fig. 16 (abstract P19).**
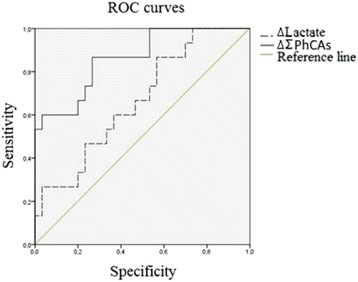
Prognostic value of PhCA and lactate clearances in high-risk patients with acute abdomen after surgery

PhCA and lactate clearances correlated with changes in SOFA scale *rs*: 0.655 and 0.531 respectively, *p < 0.05*. Logistic regression equation was formulated for each of the clearances (−2log; *p*): PhCAs (35.106;*<0.001*), lactate (52.252;*0.025*). So the proportion of true predicted solutions with the usage of the model based on PhCA clearance was 82 %, while lactate model gave only 73 %. Logistic regression determined that the treatment should be considered as effective if PhCA level reduced by 27 % or more on the third day (in this case risk of fatal outcome is lower than 10 %) and not effective if PhCA level increased more than 60 % from the initial level (as evidenced by a high risk of mortality >30 %). The prognostic value based on the clearance was more significant for PhCAs [AUC 0,862 (p < 0.001)] then for lactate [AUC 0.667 (p = 0.071)], Fig. 16.

**Conclusions**

Essentially new data suggest a promising use of PhCA clearance for the assessment of the ICU treatment efficiency.

Supported by Russian Science Foundation Grant №15-15-00110.

**References**

1. Khodakova A., Beloborodova N.: Microbial metabolites in the blood of patients with sepsis . Critical Care 2007, 11(Suppl 4):P5

2. Rogers A.J., McGeachie M., Baron R.M., Gazourian L., Haspel J.A.., Nakahira K., Fredenburgh L.E., Hunninghake G.M., Raby B.A., Matthay M.A.., Otero R.M., Fowler V.G., Rivers E.P., Woods C.W., Kingsmore S., Langley R.J., Choi A.M: Metabolomic derangements are associated with mortality in critically ill adult patients. PLoS One 2014, 9(1):e87538.

3. Beloborodova N., Moroz V., Osipov A., Vlasenko A., Bedova A., Pautova A., Sarshor Yu.: Prognostic value of phenyl carboxylic acids level in patient serum with acute abdomen. Shock 2015, 44 (Suppl 2):13.

4. Moroz V.V., Beloborodova N.V., Osipov A.A., Vlasenko A.V., Bedova A.Yu., Pautova A.K.: Phenylcarboxylic acids in the assessment of the severity of patient condition and the efficiency of intensive therapy in critical care medicine. General Reanimatology 2016, 4: in press.

5. Moroz V.V., Beloborodova N.V., Bedova A.Yu, Revel’skii A.I., Getsina M.L., Osipov A.A., Sarshor Yu N., Buchinskaya A.A., Olenin A.Yu.: Development of Methods of the Gas Chromatographic Determination of Phenylcarboxylic Acids in Blood Serum and their Adaptation to Clinical Laboratory Conditions. J Anal Chem 2015, 70(4):495–501.

**Table 6 (abstract P19).** Dynamic of PhCAs, lactate and SOFA depending on the outcome, Me (25-75 %)

Characteristic	Survived patients (n = 30)		Died patients (n = 15)	
	On ICU admission	After 48 hrs. of ICU treatment	On ICU admission	After 48 hrs. of ICU treatment
PhCAs, μM	5,5 (3,7-8,1)	4,5 (3,5-7,2)	21,6 (10,2-47,0)	75,4 (43,0-99,2)
Lactate, mmol/l	1,3 (1,0-2,1)	1,2 (0,9-1,7)	3,1 (2,5-3,9)	3,8 (2,2-10,4)
SOFA, scores	2 (1–5)	1 (0–3)	5 (4–8)	8 (7–10)

## P20 CETPI defines its function as a novel plasma LPS-binding protein with implications in the treatment of septic shock

### Jaime Mas-Oliva^1^, Victor García-González^2^

#### ^1^Instituto de Fisiología Celular, Universidad Nacional Autónoma de México, Cd. de México, México; ^2^Facultad de Medicina, Universidad Autónoma de Baja California, Mexicali, México

##### **Correspondence:** Jaime Mas-Oliva (jmas@ifc.unam.mx)

**Background**

The cholesteryl-ester transfer protein isoform exclusively expressed in the small intestine (CETPI) and present in human plasma, although discovered by our group a few years ago, it had been waiting for a functional description and a plausible role of physiological relevance [1]. Unlike the cholesteryl-ester transfer protein (CETP), CETPI does not present exon 16 and 54 bases contained in intron 15 form part of the new mRNA replacing the 24 C-terminus residues present in CETP with a sequence of 18 residues containing a high concentration of prolines and positively charged amino acids [1]. Now, the present study introduces CETPI as a new protein with the potential capability to recognize, bind and neutralize lipopolysaccharides (LPS) in circulation during septic shock. Employing several peptides derived from the C-terminus domain of CETPI we demonstrate their property not only to interact with several LPS serotypes but also to displace LPS bound to the surface of cells [2].

**Results**

Peptide VSAK derived from the last 18 residues of CETPI protects against the cytotoxic effect of LPS upon macrophages, hepatocytes and microglial cells, does not show cytotoxicity by itself, and prevents against the expression of pro-inflammatory cytokines and the generation of oxidative stress [2]. For many years, the phenomenon of recognition of bacterial LPS as components of the cell membrane of Gram-negative bacteria carried out by the innate immune system was thought to involve simple pathways if compared to the acquired immune system. Nowadays this view has dramatically changed since complex pathways involving multiple receptors and protein-protein interactions have been described. Nevertheless, despite the efforts made to counteract what it is known as the inflammatory disequilibrium syndrome and overall the outcome of septic shock, current therapeutic possibilities mainly involving inflammation and metabolic control and antibiotic treatment, have not changed for many years. Therefore, since CETPI efficiently binds LPS *in vitro* and it is overexpressed in small intestine cell cultures and proved to be present in human plasma, the efficacy of the administration of peptide VSAK against LPS cytotoxicity was studied *in vivo*. Employing a septic shock model in rabbits, we demonstrate that the infusion of peptide VSAK presents the ability to protect against the deleterious effects of LPS and the property of reducing the presence of TNFα in plasma [2, 3].

**Conclusions**

CETPI presents itself as a new protein that undoubtedly will advance the possibilities to better understand and treat the dangerous effects of LPS present during the treatment of sepsis caused by Gram-negative bacteria and its common consequence, a septic shock condition.

**References**

1. Alonso AL, Zentella-Dehesa A and Mas-Oliva J: Characterization of a naturally occurring new version of the cholesterol ester transfer protein (CETP) from small intestine. Mol Cell Biochem 2003, 245:173–182.

2. García-González V, Guitiérrez-Quintanar N and Mas-Oliva J: The C-terminal domain supports a novel function for CETPI as a new plasma lipopolysaccharide-binding protein. Sci Rep 2015, 5:16091; doi: 10.1038/srep 16091.

3. Mas-Oliva J, Gutiérrez-Quintanar N and García-González V. Peptides derived from the C-terminal domain of CETPI as blocking molecules against the cytotoxic effect induced by lipopolysaccharides in septicemia and septic shock. Patent application 2014, PCT/MX2014/000087.

## P21 Highly multiplexed molecular pathogen ID followed by phenotypic AST from whole blood using a novel fully automated system

### Jonas Jarvius^1^, Johan Mörck^1^, Ylva Molin^1^, Richard Kroon^1^, Markus Klintstedt^1^, Martin Sundqvist^2^, Charlotta Göransson^1^

#### ^1^Q-linea AB, Uppsala, Sweden, ^2^ Department of Laboratory Medicine, Clinical Microbiology, Faculty of Medicine and Health, Örebro University, Sweden

##### **Correspondence:** Jonas Jarvius (charlotta.goransson@qlinea.com)

**Background**

To address the need of more rapid diagnosis of potential pathogens causing sepsis or septic shock in patients, Q-linea is developing a fully automated, high-throughput diagnostic platform, ASTrID®.The system will perform both molecular pathogen identification and phenotypic antibiotic susceptibility testing (AST), directly from whole blood, delivering ID after four hours and AST in additional six. The pathogen panel will cover 95 % of relevant pathogens including 33 unique pathogens and 10 groups, as well as 11 resistance markers. The panel of antibiotic substances will contain 30 antibiotics and bacterial growth or inhibition reported as Minimum Inhibitory Concentration values.

**Materials/Methods**

In an ongoing study clinical blood samples are collected from patients suspected of having sepsis attending the Infectious Disease ER, University hospital of Örebro, Sweden. In addition to the standard 4 blood culture flasks drawn per patient for routine diagnostics, an extra, 5–10 ml, blood sample were taken for the present study. Pathogen ID and AST analysis was performed in a prototype ASTrID system, starting with target identification through a proprietary nucleic acid amplification reaction. Highly specific and selective padlock probes forming circularized DNA strands [1,2] are amplified via rolling-circle amplification (RCA) [3] and subsequent circle-to-circle amplification (C2CA) [4]. The resulting RCA products are labelled with fluorescence and detected on a microarray. The proprietary growth-based AST analysis was performed on a subset of clinical isolates spiked in blood (inoculum consistent with 10 CFU/ml blood).

**Results**

Of the clinical samples collected, 32 samples identified as positive in normal clinical routine diagnostics were selected and are presented in this study. Pathogen identification direct from patient in ASTrID achieved sensitivity of 96 % and specificity of 100 %, compared to conventional analysis on positive blood culture flasks. When compared with broth microdilution, the ASTrID AST results showed 99 % essential agreement and 99 % categorical agreement. Overall, the results were highly correlated with traditional technologies and EUCAST guidelines.

**Conclusion**

With ASTrID, both pathogen identification and antibiotic susceptibility testing can be done within 10 hours from blood draw. This dramatically shortens the time to adjusted treatment. The molecular pathogen identification method allows high multiplex, high sensitivity testing. The phenotypic antibiotic susceptibility profiling method developed reports reliable data directly from patient samples, without the need to wait for positive blood cultures. The new ASTrID platform from Q-linea has the potential to become a crucial tool against the global challenge of antibiotics resistance, helping to save lives and reduce healthcare costs.

**References**

1. Nilsson M, Malmgren H, Samiotaki M, Kwiatskowski M, Chowdary BP, Landegren U: Padlock probes: circularising oligonucleotides for localized DNA detection. Science, 1994 Sep 30; 265(5181): 2085–8

2. Hardenbol P, Banér J, Jain M, Nilsson M, Namsaraev EA, Karlin-Neumann GA, Fakhrai-Rad H, Ronaghi M, Willis TD, Landegren U, Davis RW: Multiplexed genotyping with sequence-tagged molecular inversion probes. Nat Biotechnol. 2003 Jun;21(6):673–8.

3. Banér J, Nilsson M, Mendel-Hartvig M, Landegren U: Signal amplification of padlock probes by rolling circle replication. Nucleic Acids Res. 1998 Nov 15;26(22):5073–8.

4. Dahl F, Banér J, Gullberg M, Mendel-Hartvig M, Landegren U, Nilsson M: Circle-to-circle amplification for precise and sensitive DNA analysis. Proc Natl Acad Sci U S A. 2004 Mar 30;101(13):4548–53.

## P22 Obligate anaerobic bacteria associated bacteremia

### Marina Sukhina^1^, Vladimir Zhukhovitskiy^2^

#### ^1^Department for Microbiology and Immunology, A.N. Ryzhikh State Scientific Center for Coloproctology, Moscow, Russia; ^2^Laboratory for Identification and Analysis of Microorganisms, N.F. Gamalei State Research Centre for Epidemiology and Microbiology, Moscow, Russia

##### **Correspondence:** Marina Sukhina (marinamari272015@gmail.com)

**Background**

Obligate anaerobic bacteria (OAB) are the dominant microflora of the human body. OAB are often the primary cause of the disease and can also become aggravating factors. OAB cause extensive tissue destruction and toxin production that result in multiorgan failure, thus OAB-associated pathology is usually very difficult to treat. Mixed behavior of anaerobic-aerobic microflora hampers the choice of antimicrobials, often OAB are antibiotic-resistant. Our aim was to assess the frequency of anaerobic bacteremia. We also evaluated antimicrobial resistance in OAB derived from blood of patients with diagnosis «sepsis».

**Materials and methods**

1300 blood samples were incubated under aerobic and anaerobic conditions on “BD Bactec Aerobic/F” and “BD Bactec Anaerobic/F” media respectively in “Bactec 9120” system (“Becton Dickinson”, USA). OAB cultures were isolated on “Blood Agar” and “Anaerobic Agar” (“Becton Dickinson”, USA) with 5 % (v/v) whole sheep blood under anaerobic conditions in anaerobic environmental chamber “Bactron™” (“Shellab”, USA). Identification and assessment of sensitivity (resistance) to antibiotics were performed by means of MALDI-TOF MS and test-systems “API 20A”, “ATB ANA”.

**Results**

57 cultures of OAB were isolated from 1300 blood samples (4,5 %). 38 OAB cultures only (67,9 % of OAB isolated) were identified using available techniques of phenotypic identification. 18 OAB cultures (32,1 %) belonged to Clostridia genus and were identified as *C. perfringens* (4 cultures), *C. sphenoides* (1), *C. acetobutylicum* (2), *C. fallax* (1), *C. clostridioforme* (1), *C. histolyticum* (1), C. difficile (3), *C. paraputrificum* (2), *C. butiricum* (2), *C. novyi* (1). Other 12 Gram-positive OAB cultures (21,4 %) were identified as *Eggerthella lenta* (1), *Eubacterium lentum* (2), *E. sabureum* (2), *Actinomyces naeslundii* (1), *Bifidobacterium breve* (1), *Propionibacterium avidum* (1), *Peptostreptococcus anaerobius* (1), *Anaerococcus prevotii* (1), *Peptococcus niger* (1), *P. saccharolyticus* (1). 7 Gram-negative OAB cultures (12,5 %) were identified as *Bacteroides fragilis* (1), *B. urealyticus* (4), Veillonella spp. (2). 30 identified OAB cultures (78,9 %) were Gram-positive and 8 (21,1 %) – Gram-negative. Among 18 non-identified OAB cultures (32,1 % of OAB isolated) 12 (66,7 % of non-identified OAB) were Gram-positive and 6 (33,3 %) – Gram-negative. 7 OAB cultures (12,5 % of OAB isolated) belonged to C*. perfringens* (2), *C. acetobutylicum* (1), *C. fallax* (1), *E. sabureum* (1), *E. lentum* (1), *P. avidum* (1) possessed resistance to metronidazole.

**Conclusions**

Growing evidence of OAB resistant to conventional antianaerobic drugs determines the necessity of monitoring resistance of anaerobes.

## P23 Antibiotic resistance patterns in pathogens derived from patients with surgical sepsis: emerging clinical challenges

### Marina A. Sukhina^1^, Igor Obraztsov^2^, Vladimir G. Zhukhovitskiy^3^

#### ^1^Department for Microbiology and Immunology, A.N. Ryzhikh State Scientific Center for Coloproctology, Moscow, Russia; ^2^Department for Clinical Immunology, D. Rogachev State Scientific and Clinical Center for Paediatric Haematology, Oncology and Immunology, Moscow, Russia; ^3^Laboratory for Identification and Analysis of Microorganisms, N.F. Gamalei State Research Centre for Epidemiology and Microbiology, Moscow, Russia

##### **Correspondence:** Igor Obraztsov (igor_obraztsov@bk.ru)

**Background**

Increasing antibiotic resistance of nosocomial pathogens impedes efficient antimicrobial therapy and significantly enhances risk for patients at early stages after operative treatment [1, 2]. Our aim was to investigate spectra of clinically relevant pathogens and their antibiotic resistance patterns to validate our current protocols of empiric antibiotic treatment.

**Materials and methods**

We enrolled 230 patients from a surgical center for coloprotology and 283 patients from a general surgical hospital who had undergone gastrointestinal surgery and then had a confirmed infectious locus along with systemic inflammatory response syndrome. 475 blood and 213 abdomen isolates were assessed by MALDI-TOF (Bruker, Germany) mass-spectrometer and “miniAPI” (“BioMèrieux”, France) analyzer.

**Results**

Both cohorts showed the same structure of blood-derived microorganisms with 54 % of Gram-positive bacteria. Gram-positive *Staphylococcus spp.* with prevalence of coagulase-negative species and Gram-negative *Klebsiella pneumoniae* dominate in both hospitals. There are statistically significant higher shares of dominant pathogens in the center for coloproctology: *Staphylococcus spp.* by 11 %, *K. pneumoniae* by 5 %; non-fermenting Gram-negative by 4 %. Resistance to penicillins was >70 %; to protected beta-lactams, fluoroquinolones and aminoglycosides was >50 %. Mean levels of resistance to cephalosporins are 79.8 % and 53.2 %, to carbapenems – 42.9 % and 52.9 % in general vs coloproctological hospitals. Share of blood-derived methicillin-resistant *Staphylococci* (MRS) equals >20 % in both hospitals, MRS showed resistance to protected beta-lactams in >90 % cases. Percentage of vancomycin-resistant *Staphylococci* is 20.0 % in coloproctology vs 1,4 % in general clinic. Respectively, blood-derived *K. pneumoniae* was resistant to cephalosporins in 63,9 % vs 78,2 %, to carbapenems – in 33.4 % vs 13.5 %, to aminoglycosides – in 68.8 % vs 47.3 %, to fluoroquinolones – in 59.6 % vs 87.2 %. Several tigecycline-resistant blood-derived *K. pneumoniae* and *Acinetobacter spp.* were isolated. Structure of abdomen-derived isolates is represented by 47,.2 % of *Enterobacteriaceae*, 40,.7 % of Gram-positive *Cocci* and 12.1 % of non-fermenting Gram-negative bacteria. General antibiotic activity against abdomen-derived microorganisms was 14.3 ± 5,.8 % higher than against blood-derived. Percentage of abdomen-derived MRS was 52.0 %, resistant to protected beta-lactams – 52.8 %, to vancomycin – 15.1 %, resistance patterns of Gram-negative microorganisms see in Table 7.

**Conclusions**

Our data shows huge burden of resistance with high portion of strains insusceptible to the last resort antibiotics including tigecycline. Predomination of *K. pneumoniae* and *Pseudomonas aeruginosa* in the structure of Gram-negative bacteraemia corresponds with the structure of abdominal isolates; higher levels of resistance in blood isolates compared to abdominal could be driven by selection of resistant strains during systemic antibiotic treatment. Thus strict microbiological monitoring is necessary for efficient antibacterial therapy.

**References**

1. Bataar O, Khuderchuluun C, Lundeg G, Chimeddorj S, Brunauer A, Gradwohl-Matis I, Duenser MW: Rate and pattern of antibiotic resistance in microbiological cultures of sepsis patients in a low-middle-income country's ICU. Middle East J Anaesthesiol 2013 Oct; 22(3):293–300

2. Salahuddin N, Amer L, Joseph M, El Hazmi A, Hawa H, Maghrabi K: Determinants of Deescalation Failure in Critically Ill Patients with Sepsis: A Prospective Cohort Study. Crit Care Res Pract. 2016 Jul; 2016:6794861. doi: 10.1155/2016/6794861.

**Table 7 (abstract P23).** Mean percentage of abdomen-derived resistant Gram-negative microorganisms, %

	*Escherichia coli*	*Klebsiella pneumoniae*	*Enterobacter spp.*	*Non-fermenting*
Penicillins	65.9	88.5	83.6	26.9
Cephalosporins	29.8	66.8	49.6	31.0
Protected beta-lactams	18.9	53.2	36.8	15.1
Carbapenems	11.5	22.1	22.7	26.4
Aminoglycosides	29.8	37.9	38.6	17.9
Fluoroquinolones	53.1	73.9	76.4	23.1

## P24 IL-35 and soluble CD14 subtype might be useful prognostic markers in critical septic patients

### Cinzia Peronace, Giovanni Matera, Luisa Galati, Aida Giancotti, Giorgio Settimo Barreca, Angela Quirino, Maria Carla Liberto, Alfredo Focà

#### Institute of Microbiology, Department of Health Sciences, University “Magna Graecia” of Catanzaro, Catanzaro, Italy

##### **Correspondence:** Alfredo Focà (cinziaperonace@hotmail.it)

**Background**

To date reliable biomarkers for prognostic evaluation of septic critical patients, are still needed. A firm pathogenetic and mechanistic association between a laboratory parameter/mediator and a clinical phenotype is a necessary assumption to adopt such parameter/mediator as a clinical biomarker [1]. CARS phase of sepsis has aroused an increasing interest among clinicians. In our previous studies, we demonstrated that sCD25 and IL-10 are reliable sepsis markers [2]. Recently presepsin (soluble CD14 subtype) has been shown to increase in plasma of septic critical subjects [3]. IL-35, a well known immunoregulatory cytokine, has been reported among novel sepsis biomarkers [4]. Aim of this study was the assessment of IL-35 and presepsin as prognostic sepsis markers in critical patients.

**Materials and methods**

For this observational prospective study, were sequentially enrolled critical patients admitted to the Unit of Intensive Care (ICU) of the University Hospital of Catanzaro Italy. For such patients admission SOFA scores were calculated. Based on 28 days survival, subjects were stratified in survivors (n = 32) and nonsurvivors (n = 17), while a group of healthy volunteers were also included as negative controls (n = 10). Moreover, clinical and microbiological data including blood culture were recorded. Plasma and serum specimens were collected at the patient admission; samples were tested for presepsin (PATHFAST Presepsin assay), IL-35 (Bio-Ocean LLC) and procalcitonin (VIDAS B.R.A.H.M.S PCT, bioMérieux, Italy). Data were subjected to statistical analysis by ANOVA plus post-hoc PLSD Fisher’s test. Selected data were processed by ROC analysis.

**Results**

**Fig. 17 (abstract P24.**
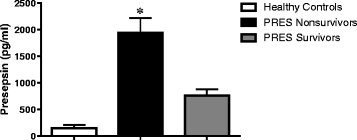
Presepsin levels in healthy controls, nonsurvivors and survivors patients at hospital admission. _*_p < 0.05 in nonsurvivors vs. survivors at the same time

At the admission SOFA scores were found significantly higher (p < 0.001) in nonsurvivor vs. survivor patients. Admission levels of presepsin were significantly more elevated (p < 0.05) (Fig. 17), in nonsurvivors vs. survivors at the same time. Presepsin concentrations were found significantly increased (p < 0.05), in blood culture-positive in comparison to culture-negative patients at the same time. PCT admission levels did not exhibit significantly different values between nonsurvivor patients and survivor subjects. However, culture-positive patients showed PCT levels at admission significantly increased vs. culture-negative (p < 0.05). Presepsin admission data from dead/alive individuals were subjected to ROC analysis, which revealed a very high accuracy and significant AUROCC (p < 0.0001). Concentrations of IL-35 were found significantly (p < 0.05) higher in nonsurvivors as well as survivors, when compared with healthy subjects.

**Conclusions**

Presepsin may substantially improve PCT contribution to clinical decisions in the patients evaluated. IL-35 appeared a promising future sepsis biomarker, which deserves further assessment. However present data indicated presepsin as a dependable prognostic tool with an elevated clinical value.

**References**

1. Ackland GL, Prowle JR: Presepsin solving a soluble (CD14) problem in sepsis? Intensive Care Med 2015, 41: 351–353

2. Matera G, Puccio R, Giancotti A, Quirino A, Pulicari MC, Zicca E, Caroleo S,Renzulli A, Liberto MC, Focà A: Impact of interleukin-10, soluble CD25 and interferon-γ on the prognosis and early diagnosis of bacteremic sistemi inflammatory response syndrome: a prospective observational study. Critical Care 2013, 17:R64.

3. Focà A, Peronace C, Matera G, Giancotti A, Barreca G.S, Quirino A, Loria MT, Settembre P, Liberto MC, Amantea B: Presepsin (soluble CD14 subtype) is a dependable prognostic marker in critical septic patients. Critical Care 2016, Volume 20, Suppl 1, P14.

4. Cao J, Xu F, Lin S, Tao X, Xiang Y, Lai X, Zhang L: IL-35 is elevated in clinical and experimental sepsis and mediates inflammation. Clinical Immunology 2015, 161: 89–95.

## P25 Sepsis following autologous hematopoietic stem cell transplantation: a predictive signature

### Yasmine Labiad^1^, Geoffroy Venton^1,2^, Céline Baier^1^, Julien Colle^1,2^, Laure Farnault^2^, Corinne Brunet^2^, Béatrice Loriod^1^, Nicloas Fernandez-Nunez^1^, Pierre Suchon^3,4^, Jean-Camille Mattei^5^, Pascal Rihet^1^, Catherine Nguyen^1^, Régis Costello^1,2^

#### ^1^Aix-Marseille Université ; INSERM, UMR1090, TAGC Campus de Luminy, Marseille, F_13288, France; ^2^Service d’Hématologie et de Thérapie Cellulaire Assistance Publique des Hôpitaux de Marseille, Centre Hospitalier Universitaire La Conception, Marseille, France; ^3^Laboratoire d’Hématologie, Assistance Publique des Hôpitaux de Marseille, Centre Hospitalier Universitaire La Timone, Marseille, France; ^4^UMR 1062 NORT, INSERM, Marseille France; ^5^UMR-911 INSERM Center for research in onco-biology and onco-pharmacology, faculté de médecine, Marseille, France

##### **Correspondence:** Yasmine Labiad (labiad.yasmine@gmail.com)

**Background**

Autologous hematopoietic stem cells transplantation (Auto-HSCT) after therapeutic intensification is based on the administration of myelosuppressive high-dose chemotherapy, followed by infusion of autologous hematopoietic stem cells in order to obtain a faster hematologic reconstitution. Hematopoietic stem cells (HSCs) infusion helps to reduce the duration of chemotherapy-induced myelosuppression and lowers the procedure-related mortality rate below 3 % [1, 2, 3]. Over the past two decades, auto-HSCT has shown remarkable development thanks to the use of hematopoietic growth factors. With few exceptions (solid tumors, autoimmune diseases), auto-HSCT is widely indicated for the treatment of hematological malignancies and is considered as a standard treatment in multiple myeloma in young patients and relapsed or refractory high-grade lymphoma (non Hodgkin and Hodgkin lymphoma). Immediately after auto-HSCT, hematologic reconstitution and infectious complications are the two major issues to monitor in transplanted patients. Besides direct toxicity of conditioning regimens, deep and prolonged neutropenia exposes patients to significant risks of infection. Though many patients will develop infectious complications after therapeutic intensification, it remains impossible to predict whether they will or not. Therefore the goal of this work was to determine and identify a sepsis predictive transcriptomic signature in patients receiving auto-HSCT.

**Materials and methods**

High throughput transcriptomic and bioinformatics analysis were performed to analyze gene expression modulation in peripheral blood mononuclear cells in 21 patients undergoing auto-HSCT for hematological malignancies.

**Results**

**Fig. 18 (abstract P25).**
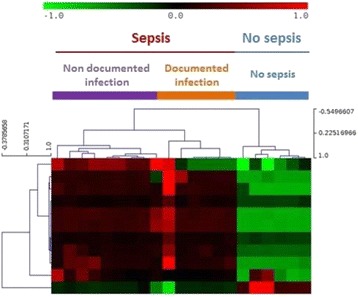
See text for description

After conditioning regimen, eleven genes were significantly differentially expressed in patients who will develop sepsis within 48 hours. Ten genes were up regulated in patients who develop sepsis compared with patients who not develop sepsis. And one gene were down regulated (Fig. 18). 100 % of patients were classified in the right group according to their gene expression and based on SVMs analysis. Transcriptomic signature may predict sepsis profiles and is more robust than the main confounding factors, such as conditioning regimen type or gender, which were therefore excluded from the analysis. In order to confirm our results, our transcriptomic signature has been validated on a 10 patient’s prospective validation cohort.

**Conclusions**

Infectious complications are a major issue in patients receiving auto-HSCT. Eleven genes are significantly differentially expressed in patients who will develop sepsis within 48 hours following the conditioning regimen. Such predictive transcriptomic signature opens up to new therapeutic strategies based on antibiotic and/or antifungal prophylaxis adapted to the specific risk profile of each patient.

**Acknowledgements**

We would like to thank INSERM, Aix-Marseille Université, Advenced Biodesign, and TGML platform supported by the France Génomique for grants. We are grateful to the patients who gave their informed consent to the use of their samples for research. We thank Laurence Borge for assistance and the use of the cell culture platform facilities (CRCM U1068, Marseille) Plateforme de Culture Cellulaire de Marseille-Luminy, bâtiment TPR2, 163 Avenue de Luminy, 13009 Marseille

**References**

[1]. Kurnick NB. Autologous and isologous bone marrow storage and infusion in the treatment of myelo-suppresson. Transfusion (Paris) 1962;2:178–87.

[2]. McFARLAND W, Granville NB, Dameshek W. Autologous bone marrow infusion as an adjunct in therapy of malignant disease. Blood 1959;14:503–21.

[3]. Clifford P, Clift RA, Duff JK. Nitrogen-mustard therapy combined with autologous marrow infusion. Lancet Lond. Engl. 1961 1;1:687–90.

## P26

**Withdrawn**

## P27 Simvastatin effects in the brain oxidative stress during experimental sepsis

### Luis Henrique A Costa^1^, Carlos Henrique R Catalão^1^, Nilton N Santos-Júnior^1^, Anderson O Souza^2^, Luciane C Alberici^2^, Maria José A Rocha^3^

#### ^1^Department of Neurosciences and Behavioral Sciences, Ribeirão Preto Medical School, University of São Paulo, Ribeirão Preto, Brazil; ^2^Department of Physics and Chemistry, School of Pharmaceutical Sciences of Ribeirão Preto, University of São Paulo, Ribeirão Preto, SP, Brazil; ^3^Department of Morphology, Physiology and Basic Pathology, School of Dentistry of Ribeirão Preto, University of São Paulo, Ribeirão Preto, Brazil

##### **Correspondence:** Luis Henrique A Costa (luis.angenendt@gmail.com)

**Background**

Previous studies have shown that during sepsis, brain oxidative stress and damage are induced by overproduction of reactive oxygen species (ROS). Although there are recent reports about the benefits of statins in experimental sepsis and endotoxemia in peripheral organs, little is known about its effect in the central nervous system. Our objective was to investigate the antioxidant properties of simvastatin and its possible neuroprotective effect during experimental sepsis.

**Materials and methods**

Male Wistar rats (250–300 g) were submitted to cecal ligation and puncture (CLP, n = 34) or remained as non-manipulated control (or naive, n = 34). Both groups were treated by gavage with simvastatin (20 mg/kg) or an equivalent volume of saline. The animals submitted to CLP were treated 4 days before and 48 h after surgery. One animal group was decapitated and blood and brain were collected to quantify plasma levels of cytokines and assess astrogliosis and apoptosis in the prefrontal cortex and hippocampus. Another group was perfused with PBS (0,01 M), and the same brain structures were dissected to analyze oxidative damage.

**Results**

The CLP-rats treated with simvastatin showed a reduction in nitric oxide (P < 0.05), IL1-β (P < 0.001), IL-6 (P < 0.01) and TBARS levels (P < 0.001) and an increase in catalase activity (P < 0.01), citrate synthase enzyme (P <0.05) and normalized GSH/GSSG ratio. In addition, the histopathological analysis showed a reduction (P < 0.001) in reactive astrocytes and caspase 3-positive apoptotic cells.

**Conclusions**

The results suggest a neuroprotective effect of simvastatin in structures responsible for the spatial learning and memory. Behavioral studies are needed to evaluate its impact on cognitive damage triggered by experimental sepsis encephalopathy.

